# Effectiveness and mechanism of metformin in animal models of pulmonary fibrosis: A preclinical systematic review and meta-analysis

**DOI:** 10.3389/fphar.2022.948101

**Published:** 2022-09-06

**Authors:** Xuanyu Wu, Xiang Xiao, Xinyu Chen, Maoyi Yang, Zhipeng Hu, Sijia Shuai, Qinwei Fu, Han Yang, Quanyu Du

**Affiliations:** ^1^ Hospital of Chengdu University of Traditional Chinese Medicine, School of Clinical Medicine, Chengdu University of Traditional Chinese Medicine, Chengdu, China; ^2^ Department of Geriatrics, Hospital of Chengdu University of Traditional Chinese Medicine, Chengdu, China; ^3^ Department of Endocrinology, Hospital of Chengdu University of Traditional Chinese Medicine, Chengdu, China

**Keywords:** pulmonary fibrosis, metformin, animal models, potential mechanisms, meta-analysis

## Abstract

**Background:** Pulmonary fibrosis (PF) is a lung disease with no curative drug, characterized by a progressive decrease in lung function. Metformin (MET) is a hypoglycemic agent with the advantages of high safety and low cost and has been used in several *in vivo* trials to treat fibrotic diseases.

**Objective:** This study aimed to explore the efficacy and safety of MET in treating PF and elaborate on its mechanism.

**Methods:** Eight databases were searched for *in vivo* animal trials of MET for PF from the time of database creation until 1 March 2022. The risk of bias quality assessment of the included studies was conducted using SYRCLE’s risk of bias assessment. Pulmonary inflammation and fibrosis scores were the primary outcomes of this study. Hydroxyproline (HYP), type I collagen (collagen I), α-smooth muscle actin (α-SMA), transforming growth factor-β (TGF-β), Smad, AMP-activated protein kinase (AMPK), and extracellular signal–regulated kinase (ERK) protein expression in lung tissues and animal mortality were secondary outcomes. Effect magnitudes were combined and calculated using Revman 5.3 and Stata 16.0 to assess the efficacy and safety of MET in animal models of PF. Inter-study heterogeneity was examined using the *I* ^*2*^ or Q test, and publication bias was assessed using funnel plots and Egger’s test.

**Results:** A total of 19 studies involving 368 animals were included, with a mean risk of bias of 5.9. The meta-analysis showed that MET significantly suppressed the level of inflammation and degree of PF in the lung tissue of the PF animal model. MET also reduced the content of HYP, collagen I, α-SMA, and TGF-β and phosphorylation levels of Smad2, Smad3, p-smad2/3/smad2/3, ERK1/2, and p-ERK1/2/ERK1/2 in lung tissues. MET also elevated AMPK/p-AMPK levels in lung tissues and significantly reduced animal mortality.

**Conclusion:** The results of this study suggest that MET has a protective effect on lung tissues in PF animal models and may be a potential therapeutic candidate for PF treatment.

**Systematic Review Registration:** https://www.crd.york.ac.uk/PROSPERO/display_record.php?RecordID=327285, identifier CRD42022327285.

## 1 Introduction

Pulmonary fibrosis (PF) is characterized by a progressive and irreversible decrease in lung function, eventually leading to respiratory failure and death. Chemical injury, drug toxicity, environmental factors, autoimmunity, and inflammatory infections are significant causes of PF pathogenesis (Thannickal et al., 2004). The most severe type of PF is idiopathic pulmonary fibrosis (IPF), which has no specific cause, with a median survival of 3–5 years after disease onset (Raghu et al., 2011; Thannickal, 2013; Travis et al., 2013). The global incidence of IPF has increased (Hutchinson et al., 2015)**,** with a high incidence among older men, especially those with chronic diseases, such as hypertension and diabetes (King and Nathan, 2017).

In recent years, researchers have begun to link structural remodeling in the lungs to abnormalities in glucose metabolic pathways (Dos Santos et al., 2018). Diabetes mellitus (DM) is a systemic metabolic disease characterized by insulin deficiency or resistance, which causes a chronic hyperglycemic state. Inflammation, oxidative stress, and vascular damage caused by hyperglycemia often damage the cardiovascular system, kidneys, retina, and other organs (Kowluru and Chan, 2007; Kashihara et al., 2010; Folli et al., 2011; Fujita et al., 2013). The lungs possess a complex capillary network and are a target organ for diabetic microvascular injury. The correlation between DM and PF has attracted the attention of researchers in recent years. Patients with DM are at a higher risk of developing PF (Kopf et al., 2018). Hyperglycemia can thicken the alveolar septa, cause PF-related pathological structural changes in the lungs, and affect lung function, ultimately resulting in PF (Zou et al., 2017). Hyperglycemia causes inflammation and oxidative stress in the body, which are closely related to the core developmental mechanisms of PF, such as extracellular matrix (ECM) deposition, fibroblast/myofibroblast proliferation, and structural disruption of the lung tissue (Willis & Borok, 2007). Similarly, in a meta-analysis, a significant increase in the prevalence of DM was demonstrated in patients with IPF compared with that in controls, suggesting a potential positive association between DM and the development of IPF (Bai et al., 2021).

There is a lack of specific therapeutic drugs for the treatment of PF. Pirfenidone and nintedanib (Raghu et al., 2015; Teague et al., 2022), approved for the treatment of PF, have been shown to reduce the decline in forced vital capacity (FVC) and deterioration in the acute phase in patients with IPF (Richeldi et al., 2011; King et al., 2014). However, current studies show that these two drugs do not cure or reverse PF (Wollin et al., 2015); they can only prevent the development or further progression of PF. To date, no drug has been able to reverse already established PF (Dos Santos et al., 2018). Only a few patients can afford pirfenidone and nintedanib because they are expensive (Teague et al., 2022). Moreover, the diagnosis of IPF and the adverse effects of these drugs are uncertain (Maher et al., 2018; Maher and Strek, 2019). Lung transplantation is the best approach to prolong survival in patients with PF (Wynn, 2011). Therefore, it is essential to identify additional ways to treat PF. In 2018, Rangarajan et al. proposed that metformin (MET) could reverse bleomycin-induced PF in animal models. As an older drug, this new therapeutic effect of MET is appealing (Rangarajan et al., 2018). Additionally, MET is cheaper and more conducive to further research **(** Diabetes Prevention Program Research Group, 2012 **)**.

MET, a biguanide derivative that acts on the liver to reduce high blood glucose levels, is a commonly used first-line drug for the treatment of type 2 diabetes mellitus (T2DM) and has been used for more than 60 years (Bailey, 2017). The central mechanism of MET action is the inhibition of mitochondrial respiratory chain complex I **(** Batandier et al., 2006 **)**, which reduces adenosine triphosphate (ATP) production, as well as decreases adenosine monophosphate (AMP) deaminase activity and increases AMP content, thereby increasing the AMP/ATP ratio (Richeldi et al., 2011) and activating AMP-dependent protein kinase (AMPK). AMPK is a heterotrimeric protein with important roles in the metabolism and regulation of glucose, lipids, and energy (Lin & Hardie, 2018). AMPK activation enables cells to switch from an anabolic to a catabolic state (Viollet et al., 2012). It can reduce and improve DM (Foretz et al., 2019), obesity (Yerevanian & Soukas, 2019), effects of aging **(** Chaudhari et al., 2020 **)**, and cancer (Coyle et al., 2016).

Recent studies have shown that MET may inverse PF. MET may inhibit the transforming growth factor-beta (TGF-β)/Smad signaling pathway by activating AMPK signaling. Treatment of PF mouse models with MET resulted in significant improvement in several fibrosis marker indicators, including hydroxyproline (HYP), α-smooth muscle actin (α-SMA), TGF-β1, type I collagen (collagen I), and fibronectin. However, the anti-PF effect of MET disappeared when the same approach was used to treat AMPKa-deficient mice, suggesting that MET may act as an anti-PF agent by activating AMPK (Rangarajan et al., 2018). MET may inhibit the mammalian rapamycin (mTOR) signaling pathway *via* AMPK, upregulate Beclin1 and light chain 3 B (LC3B), downregulate p62 protein, and activate autophagy. It also inhibits the inflammatory cytokines TGF-β1, tumor necrosis factor-α (TNF-α), and interleukin-1β (IL-1β) and reduces alveolar inflammation. Elevated E-cadherin (E-Cad) levels decrease α-SMA and vimentin and inhibit the epithelial–mesenchymal transition (EMT) process (Li et al., 2021b). The anti-PF effect of MET involves the inhibition of reactive oxygen species (ROS) and lipid peroxidation product malondialdehyde (MDA), which catalyzes the expression of antioxidant proteins to inhibit intracellular oxidative stress and other pathways (Cheng et al., 2021).

**FIGURE 1:**
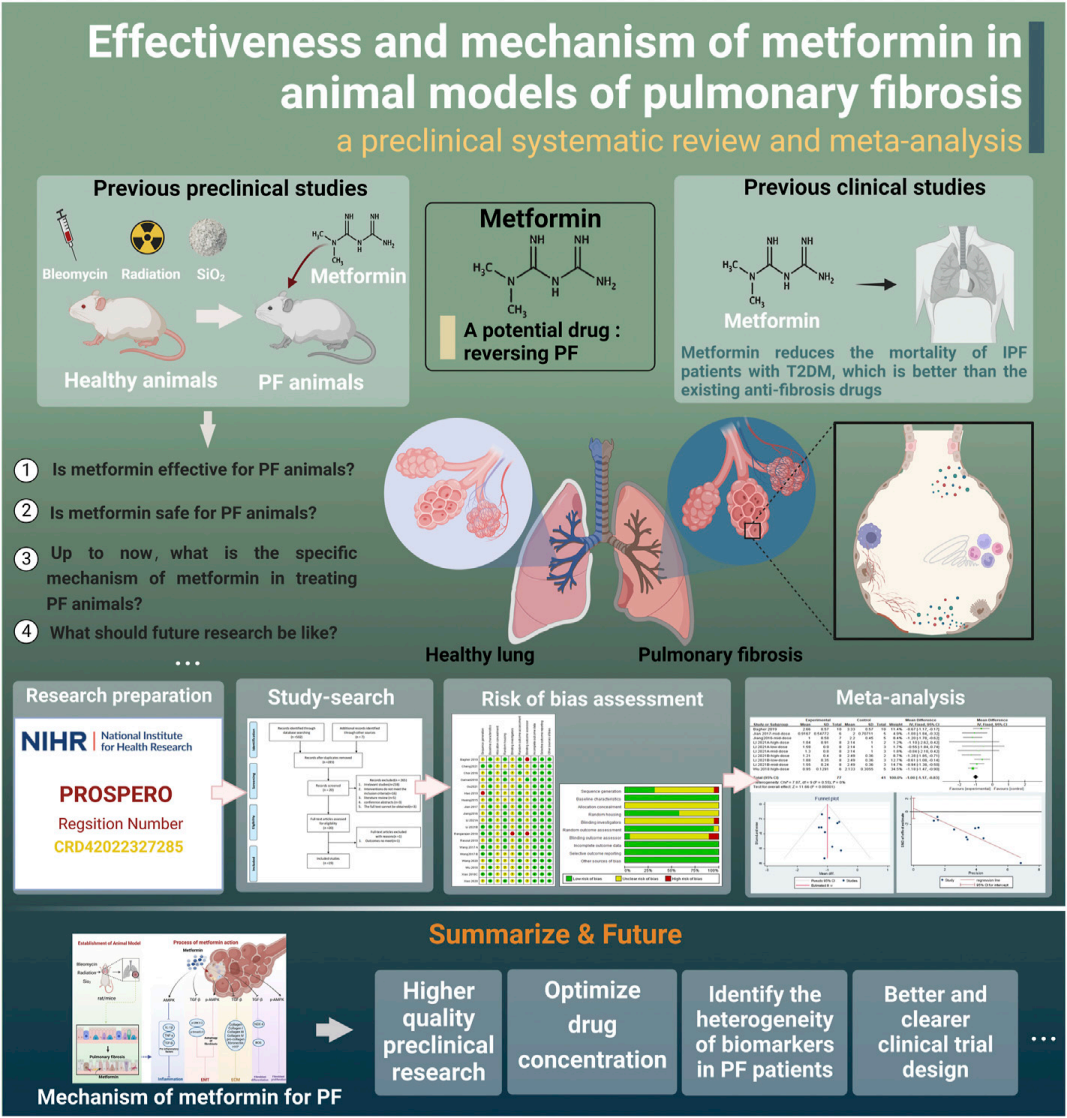
Research roadmap (created using BioRender.com).

We aimed to determine if MET could reverse PF. Our hypothesis was positive, and we believe that MET is a potential anti-fibrosis drug that needs to be developed and studied further. However, the efficacy and safety of MET to treat PF in animals is still uncertain and lacks sufficient evaluation; therefore, it is necessary to conduct a systematic evidence-based evaluation. Our goal was to systematically evaluate and meta-analyze the effectiveness and safety of MET in treating PF in animal models, determine the effect of MET on the related factors in the process of PF, and further explore its cellular and molecular mechanisms. A research roadmap is in Figure 1.

## 2 Materials and methods

This review was based on the Preferred Reporting Items for Systematic Reviews and Meta-analyses (PRISMA) statement checklist (Page et al., 2021). The protocol was registered in PROSPERO (registration number: CRD42022327285).

### 2.1 Data sources and search strategy

Eight databases were searched from the time of establishment to 1 March 2022: PubMed, EMBASE, Web of Science, Cochrane Library, China National Knowledge Infrastructure, Chinese Biomedical Database, VIP Database, and Wanfang Database. The abovementioned databases were searched with MET (e.g., metformin, dimethylguanylguanidine, and metformin HCl), pulmonary fibrosis (e.g., pulmonary fibrosis, alveolitis, fibrosis, and idiopathic pulmonary fibrosis), and animal models (e.g., models, animal, animal model, and experimental) as the main keywords, and the references of the included studies were searched to obtain more available studies. The language used was limited to Chinese and English. A detailed search strategy for each database is provided in Supplementary Material S1.

### 2.2 Eligibility criteria and literature screening

The inclusion and exclusion criteria were predefined for the meta-analysis (Table 1) (Sun et al., 2021). Two investigators (XY-C and QW-F) independently screened the literature using EndNote X9 software. After eliminating duplicate literature, investigators excluded the literature that did not meet the inclusion criteria by reading the titles and abstracts and confirmed the final included literature by reading the full articles. In the event of disagreement, a third investigator (H-Y) arbitrated.

**TABLE 1:** Inclusion and exclusion criteria.

Principles	Inclusion criteria	Exclusion criteria
Animals	1. There is no restriction on the race, category, weeks of age, and sex of the included animals. Any animal previously used for pulmonary fibrosis (PF) modeling will be included	1. Not an animal model of PF.
		2. Animals that have not been previously used to establish PF models
Interventions	2. Animal models of pulmonary fibrosis in the experimental and control groups are modeled using bleomycin, radiation, or silica	3. Not using bleomycin, radiation, or silica to establish PF animal models
	3. The experimental group received metformin/metformin hydrochloride. The control group was given saline or no measure of treatment	4. Treatment with metformin-based prescriptions or in combination with other drugs
	4. The route of administration is not restricted	5. Experimental design without a control group
Type	5. Randomized controlled animal experiments (*in vivo* studies) are required	6. Case reports, clinical trial studies, abstracts, editorials, reviews, conference abstracts, duplicate data, and incomplete text
Outcomes	6. (1) The primary outcome indicators are the histological effect of metformin on the degree of PF and pulmonary inflammation in animal models, using the pulmonary inflammation score to represent the degree of pulmonary inflammation and the pulmonary fibrosis score to represent the degree of PF.	7. No predetermined outcome indicators or available data
	(2) Secondary outcome indicators are the effects of metformin on fibrosis-related proteins *in vivo* in animal models of PF, including TGF-β, HYP, α-SMA, collagen I, AMPK, and extracellular signal–regulated kinase animal mortality	8. The literature does not use numerical quantification, and quantitative data are not available through the literature

### 2.3 Data extraction

Two investigators (XX and SJ-S) independently extracted detailed data for the included studies using Excel 2019 software, including the first author, year of publication, modeling method and route, animal details, MET manufacturer batch, MET treatment regimen, narcotic drug use, ethical statement status, intervention site, primary/secondary outcomes, and between-group differences. Experimental data were uniformly recorded using the mean and standard deviation (SD), and if only standard error (SEM) was provided, the raw data were converted to standard deviation according to statistical principles. If the literature presented the experimental results graphically, Origin 2018 was used to extract data from the images, and multiple readings were obtained to reduce errors. If the literature lacked data or was reported unclearly, XY-W attempted to contact the corresponding author; if the original data were still unavailable, XY-W excluded the literature. After completing data extraction, the two investigators crosschecked the results. Disagreements were marked, summarized, discussed, and resolved in a meeting. The study was excluded if any essential results were not available.

### 2.4 Assessment of risk of bias

Two investigators (MY-Y and ZP-H) used SYRCLE’s risk-of-bias quality assessment tool for animal experiments (Hooijmans et al., 2014). Risk of bias and methodological quality assessments of the included studies were performed. A score of 1 was assigned during the assessment process when the investigator judged an entry to be at low risk of bias, with a maximum score of 10 for one piece of literature. Disagreements were resolved by consensus or arbitration between the two authors (HY and QY-D).

### 2.5 Statistical analysis

For data processing of the included studies, one study contained multiple subgroups of MET doses, which were treated as independent experiments for this meta-analysis. The sample size of the control group for these independent studies was extracted by dividing the sample size of the control group by the number of experimental groups to avoid artificially increasing the sample size of the control group and further improving the accuracy of the study (Lin et al., 2020). In addition, the doses administered were pre-classified based on the inclusion of experiments to facilitate further subgroup analysis. The doses of administered MET were divided into three groups (0 < low-dose group <150 mg/kg, 150 mg/kg ≤ medium-dose group <300 mg/kg, and 300 mg/kg ≤ high-dose group ≤500 mg/kg) according to previous studies (Gamad et al., 2018; Wu, 2018; Hao et al., 2019; Li et al., 2021a; Li et al., 2021b; Cheng et al., 2021). 

RevMan 5.3 and Stata 16.0 were used for data analysis in this meta-analysis. The lung tissue fibrosis score, inflammation score, and fibrosis-related proteins were analyzed as continuous variables. The mean difference (MD) and 95% confidence interval (CI) were used for the overall effect size comparison if the effect size units were the same or the assay was the same between studies. Moreover, the standardized mean difference (SMD) and 95% CI were used if the effect size units were different or if the assay differed between studies. Animal mortality was used as a dichotomous variable, and relative risk (RR) and 95% CI were used as the effect analysis statistics. Differences were considered statistically significant at *p* < 0.05.

Heterogeneity between studies was tested using the *I* ^*2*^ or Q-test. Heterogeneity was considered nonsignificant when *p* > 0.1 or *I* ^*2*^ ≤ 50. When *p* < 0.1 or *I* ^*2*^ > 50, heterogeneity was considered significant, and sensitivity analysis was performed to determine the stability of the results. If any one piece of literature was found to have a more significant effect on inter-study heterogeneity, the effect sizes were combined after excluding that piece of literature. If there was excessive heterogeneity in important outcomes (i.e., inflammation score, fibrosis score, and TGF-β levels) and the cause could not be found after sensitivity analysis, subgroup analysis or meta-regression was used to further search for the source of heterogeneity. Dosage is the predetermined factor of subgroup analysis (Lin et al., 2020) because it may be an important factor affecting the effect (Garber et al., 1997). Each subgroup included at least two studies. For meta-regression, eight variables were analyzed: modeling method, route of administration, time of administration, animal sex, animal type, dosage, year of publication, and manufacturer of MET. The effect of other possible factors on inter-study heterogeneity was explored in the analysis, and variables were considered sources of heterogeneity when *p* < 0.05. When heterogeneity was substantial and could not be eliminated, a random effects model was used instead of a fixed effects model. When more than ten studies were included, funnel plotting and observation were performed using Stata 16.0 with Egger’s test (Sun et al., 2021). Publication bias was assessed by observing whether the funnel plot was symmetrical and the *p*-value of Egger’s test. When the funnel plot was significantly asymmetrical and Egger’s test *p* < 0.05, the difference was considered significant, i.e., having publication bias.

## 3 Results

### 3.1 Inclusion of study selection

**FIGURE 2:**
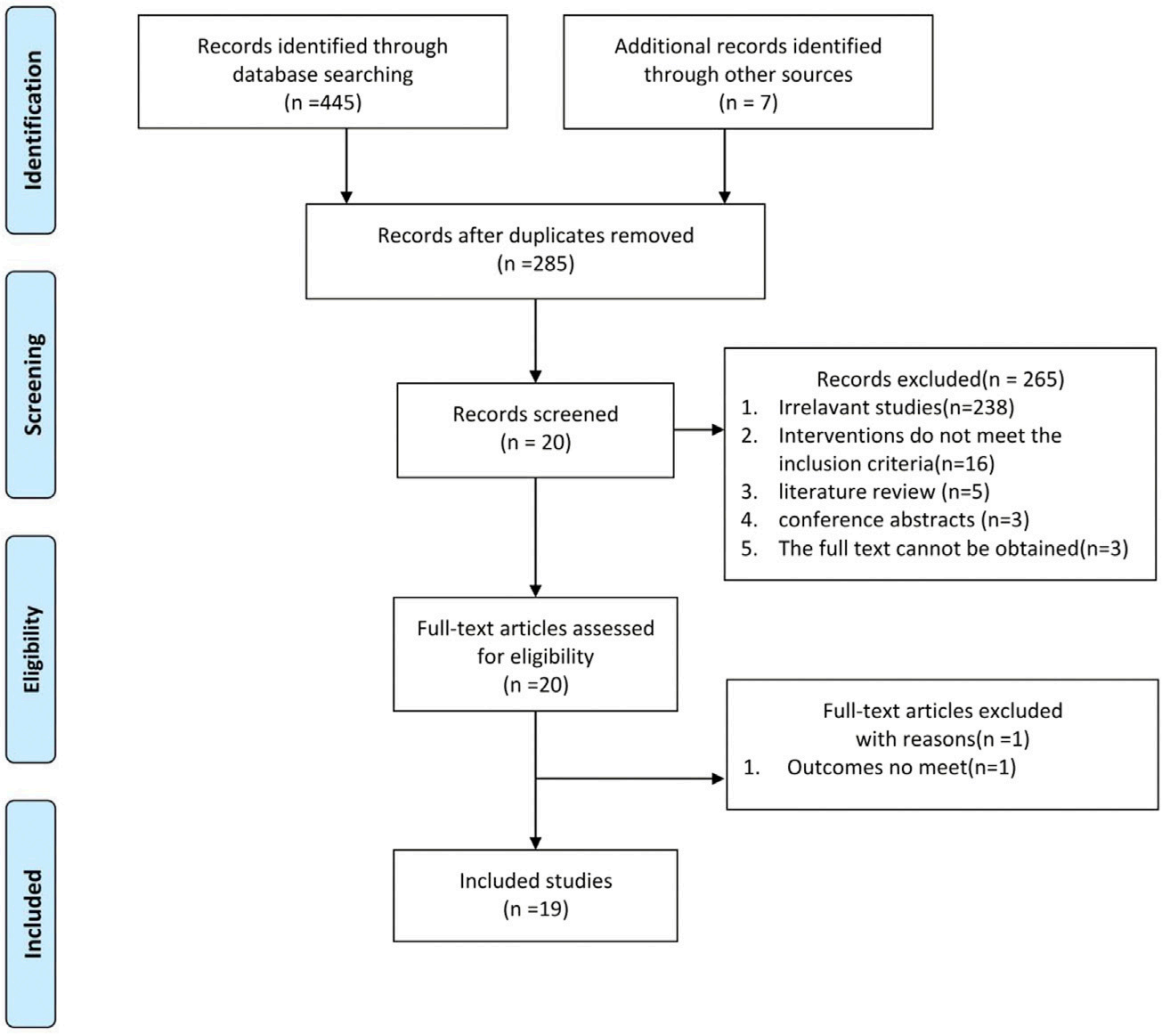
Flow diagram of the study-search process.

Through a preliminary search of eight databases, 445 studies were identified. In addition, seven articles from our records met the requirements for this meta-analysis but did not appear in the search results, and we included those (Xavier et al., 2021). After removing duplicate studies, 285 articles remained, and we performed title and abstract readings to exclude 265 articles, including 238 irrelevant studies, 16 studies whose interventions did not meet inclusion requirements, five literature reviews, three conference abstracts, and three articles for which the full text was not available. The complete manuscripts of the 20 screened studies were then read, and one manuscript with an outcome that did not meet the requirements was excluded (Kheirollahi et al., 2019), resulting in 19 remaining studies (Huang, 2015; Choi et al., 2016; Jiang 2016; Jian 2017; Wang J. et al., 2017; Wang Y. et al., 2017; Gamad et al., 2018; Rangarajan et al., 2018; Wu 2018; Xiao et al., 2018; Farhood et al., 2019; Hao et al., 2019; Yahyapour et al., 2019; Wang et al., 2020; Xiao et al., 2020; Li et al., 2021a; Li et al., 2021b; Cheng et al., 2021; Gu et al., 2021). Six studies included multiple dose groups. Two independent trials were conducted with two levels of dosing (Choi et al., 2016; Hao et al., 2019), and four independent trials had three levels of dosing [Cheng et al., 2021; Gamad et al., 2018; Li et al., 2021a; Li et al., 2021b] out of the 29 independent studies. A flow chart of literature selection is shown in Figure 2.

### 3.2 Study characteristics

The characteristics of all the included studies are summarized in Supplementary Tables S2 and S3. Supplementary Table S2 shows the basic information of the included studies, and Supplementary Table S3 shows the modeling and drug treatment of the included studies. A total of 368 experimental and control animals were included in this meta-analysis. Eight of the 19 studies used mice, and the types of mice used included wild-type, C57BL/6, and NMRI. A total of 11 studies used rats; of these, eight used Sprague-Dawley rats, and three used Wistar rats. Of the studies that have elaborated on the sex of the animals, three used female, 15 used male, and one did not report sex. Only two studies have reported animals used as adults (Wang Y. et al., 2017; Gamad et al., 2018).

All included studies used three modalities, four used irradiation, three used silica particle tracheal injection, and 12 used bleomycin tracheal injection. Among the 12 studies using bleomycin, two did not report the companies of bleomycin manufacture, one was purchased from Fresenius Kabi Oncology Limited, India, one was from Hanhui Pharmaceuticals Co., Ltd., Zhejiang, China, four were from Zhejiang Hisun Pharmaceutical Co., Ltd., one was from Thermo Fisher Scientific, and three were from Nippon Kayaku, Takasaki, Japan. Modeling drugs may be an important factor affecting the results of this study. The conditions for subgroup analysis were not met, and a descriptive analysis was conducted for comparison. After modeling, the pathological manifestations of these 12 studies mentioned thickening of the alveolar septum, infiltration of inflammatory cells, and an increase in collagen fibers. Research on fibrosis and inflammation scores has also been reported. There were significant differences in fibrosis and inflammation scores between the two groups compared with that in the blank control group (*p* < 0.05). Therefore, it can be assumed that the bleomycin used in the literature included in this study were all positive in terms of their modeling effect.

All studies controlled the duration of MET treatment between 2 and 5 weeks, limiting the treatment duration to this range. MET used in the included studies was obtained from six different manufacturers, including Sino-American Shanghai Squibb Pharmaceuticals Ltd. (6), Sigma Aldrich Corporation, United States (5), Tehran Chemie Pharmaceutical Company (2), Beyotime Institute of Biotechnology, Shanghai Sine Pharmaceutical Laboratories Co., Ltd., and Cipla Pharmaceutical (1 each). Three studies did not specify the drug manufacturer used.

Drug dose is an important factor influencing the efficacy of MET (Garber et al., 1997). In the included studies, MET was administered at doses ranging from 65 to 500 mg/kg. The 29 independent studies included 11, nine, and nine studies in the low-, medium-, and high-dose groups, respectively. In the literature by Choi et al., 2016, two low-dose groups, 50 and 100 mg/kg, were present in the same study; both were considered low-dose groups, but the later combined effect sizes were distinguished by naming them Choi 2016 (50 mg/kg) and Choi 2016 (100 mg/kg).

Adverse reactions (i.e., animal deaths during the experiment) were reported in four of the 19 studies. Researchers who use animals in pursuit of academic goals must adhere to ethical principles. Among the included studies, 11 reported ethical considerations, whereas eight did not. A total of 13 studies reported the use of anesthetics, of which six used pentobarbital sodium, four used chloral hydrate 10%, two used a combination of ketamine and xylazine, one study used isoflurane, and six did not report anesthesia. Of note, four studies reported ethical statements but did not report the use of anesthetics.

### 3.3 Risk of bias and quality of included studies

**FIGURE 3:**
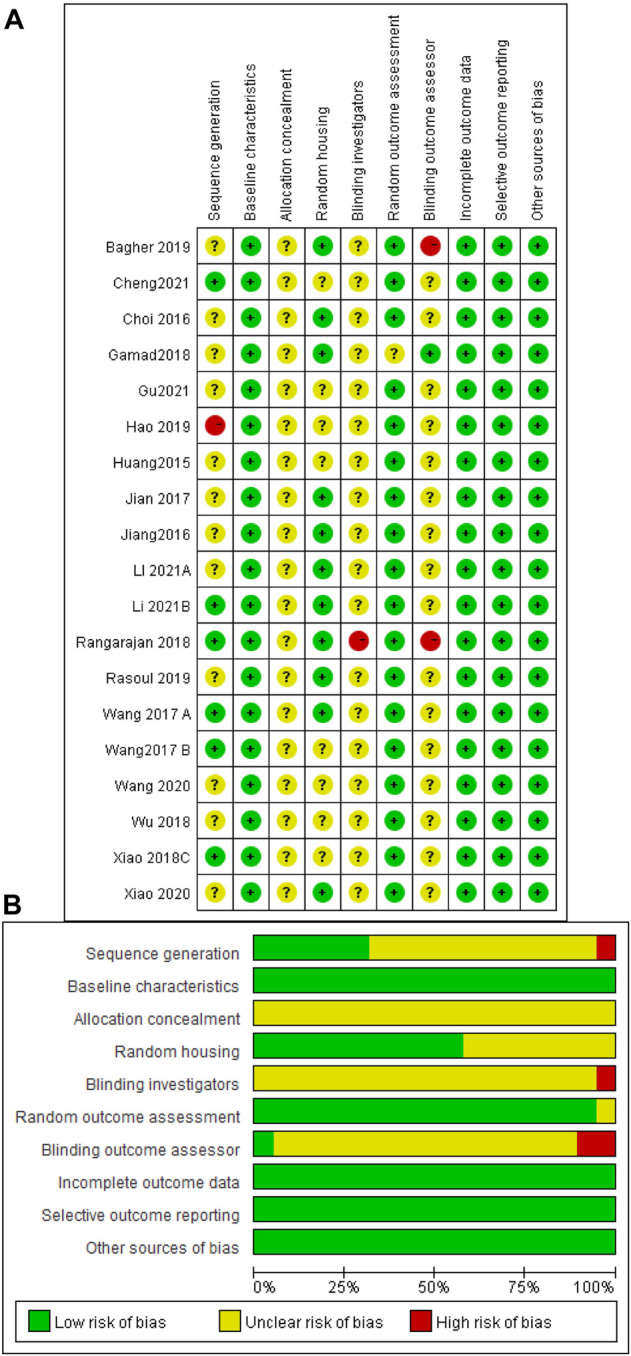
Risk of bias assessment table. Assessment of literature quality results obtained through the risk of bias by SYRCLE based on Cochrane tools. **(A)** Risk of bias summary diagram; review of authors’ judgments for each risk of bias item for each included study. **(B)** Risk of bias diagram; overview of authors’ judgments for each risk of bias item, expressed as a percentage of all included studies.

The risk of bias and quality of all studies were assessed and scored according to SYRCLE’s risk of bias tool (Hooijmans et al., 2014) to obtain the included literature scores (Figure 3). The risk of bias scores of the included studies ranged between 5 and 7, with a mean of 5.9. Six studies were rated as low in terms of random sequence generation because they described the method of random sequence generation in detail. All studies were rated low for baseline characteristics because of the detailed description of animal characteristics and ensuring that animals were similar at baseline. When assessing allocation concealment, all studies showed unclear risk, and none described in detail whether animals were concealed at allocation. For the assessment of animal placement randomization, 11 studies were rated as low risk because they described in detail the consistency and randomization of the housing environment of the experimental animals. None of the included studies mentioned the animal keeper or the investigator performing blinding, and one study was rated as high risk because it did not describe the experimental procedure. One study was rated as having unclear risk in the evaluation of randomized outcomes because it did not state whether animals were randomly selected for outcome assessment. One study described the specific method of blinding the outcome assessor, while the remaining studies did not. Two of the remaining studies were judged to be at high risk because they explicitly did not blind the outcome assessors. None of the studies had incomplete data, selective outcome reporting, or other sources of bias.

### 3.4 Outcomes

#### 3.4.1 Metformin reduced inflammation scores in lung tissues of pulmonary fibrosis animals

**FIGURE 4:**
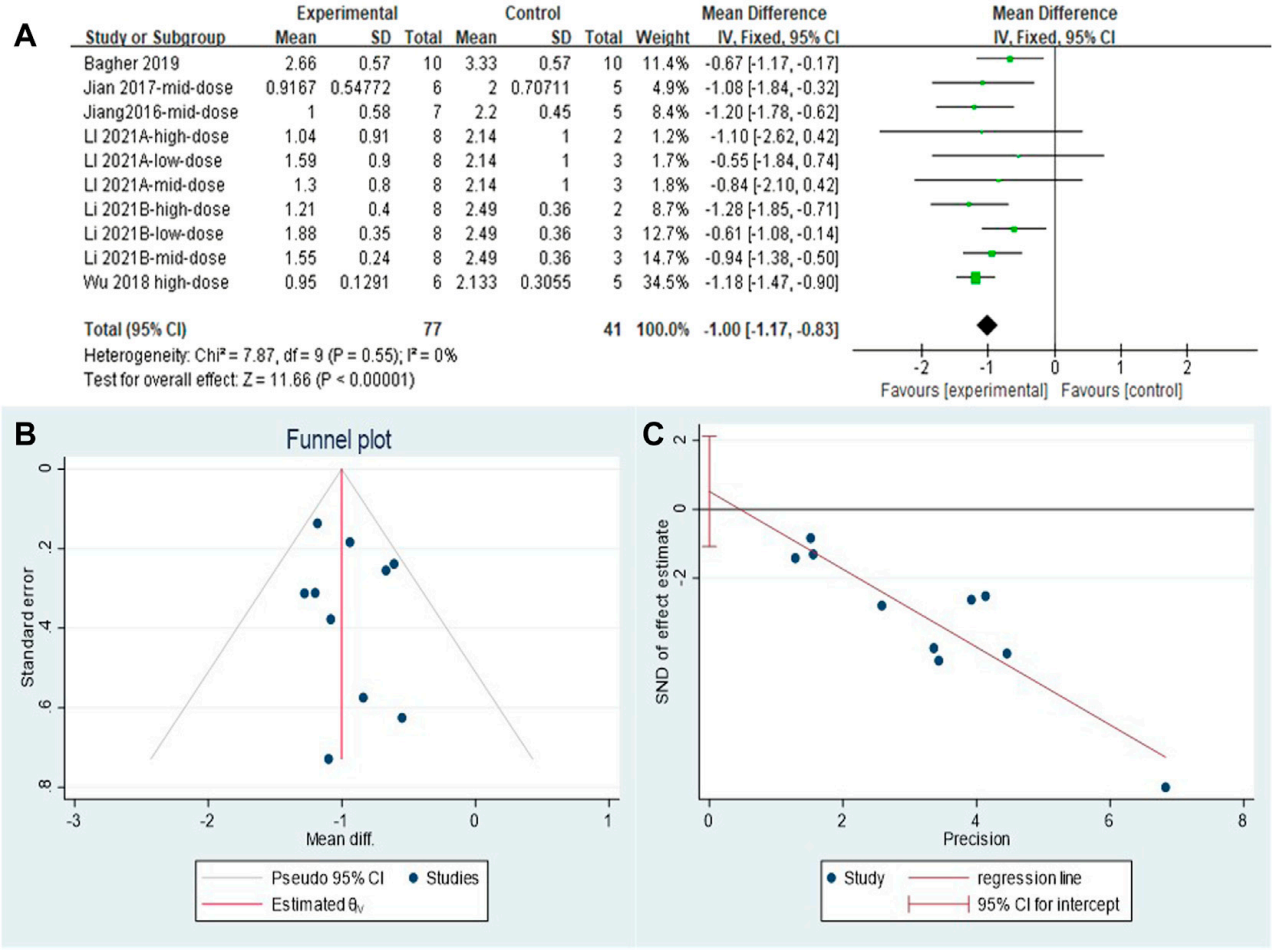
Effect of MET on inflammation scores of lung tissues in PF animals. **(A)** Forest plot of inflammation scores; **(B)** Funnel plot of inflammation scores; **(C)** Egger’s test for inflammation scores.

The effect sizes were combined for inflammation scores in the lung tissue of animals in ten independent experiments (Figure 4A). MET use significantly reduced lung inflammation scores in the treatment groups compared with that in controls [SMD = −1.00, 95% CI (−1.17, −0.83), *p* < 0.00001]. *I* ^*2*^ = 0%, and there was no heterogeneity between studies. The funnel plot symmetry (Figure 4B) and Egger’s test (Figure 4C) indicated no publication bias (*p* > 0.05).

#### 3.4.2 Metformin reduced fibrosis scores in lung tissues of pulmonary fibrosis animals

**FIGURE 5:**
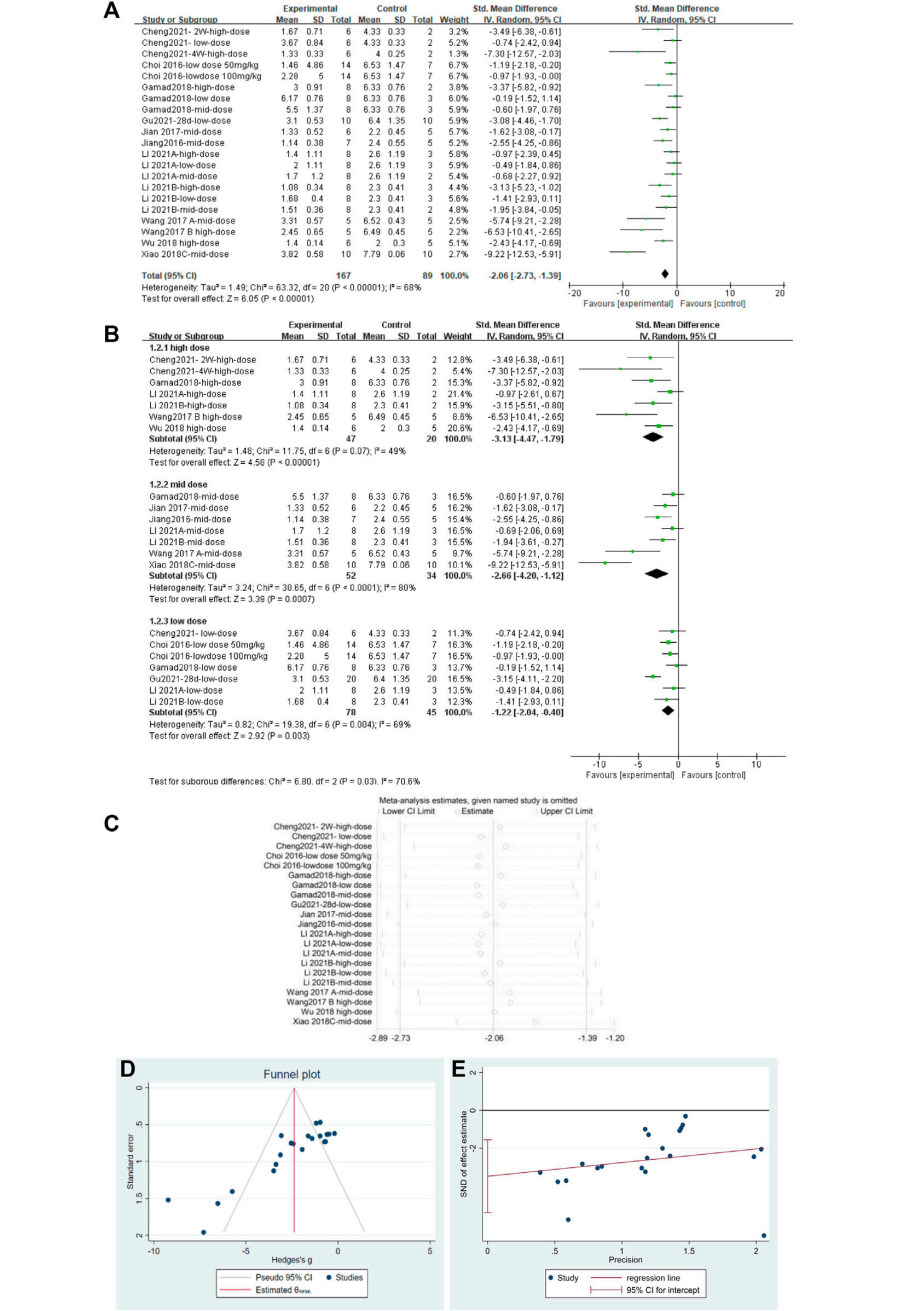
Effect of MET on fibrosis scores of lung tissues in PF animals. **(A)** Forest plot of fibrosis scores; **(B)** Forest plot of fibrosis scores (subgroups); **(C)** Sensitivity analysis of fibrosis scores; **(D)** Funnel plot of fibrosis scores; **(E)** Egger’s test for fibrosis scores.

A total of 23 independent studies reported fibrosis scores. The studies by Rasoul (2019) (Yahyapour et al., 2019) and Bagher (2019) (Farhood et al., 2019) were not included in the effect size analysis because their values did not meet the requirements for continuous variable effect size calculation. The effect sizes were combined for lung tissue fibrosis scores in animals from the 21 independent trials (Figure 5A), and the overall effect of MET was a significant reduction in lung fibrosis scores in the treatment groups compared with that in controls [SMD = –2.06, 95% CI (–2.73, –1.39), *p* < 0.00001] with high inter-study heterogeneity. Sensitivity analysis was subsequently performed (Figure 5B), excluding data from independent experiments; the heterogeneity did not decrease significantly, demonstrating the reliability of the results. We conducted subgroup analyses of the 21 trials and combined the effect sizes. The results showed a significant decrease in PF scores for all three dose levels of MET. However, subgroup analysis (Figure 5C) revealed a high degree of heterogeneity between high-dose, mid-dose, and low-dose subgroups, with *I* ^*2*^ values of 49, 80, and 69%, respectively. In addition, funnel plots (Figure 5D) and Egger’s test (Figure 5E) indicated a publication bias (*p* < 0.05). To further explore the heterogeneity between studies, a meta-regression analysis of factors contributing to heterogeneity (modeling method, route of administration, time of administration, animal sex, animal type, dosage, year of publication, and manufacturer of MET) was performed (Table 2). However, the results showed that none of the abovementioned factors was a significant source of heterogeneity (*p* > 0.05).

**TABLE 2:** Meta-regression analysis of the effect of MET on lung tissue fibrosis scores in PF animals.

Sources of heterogeneity	*P*>|t|	(95% Conf. Interval)	
Modeling Method	0.390	−1.573	3.696
Route of Administration	0.832	−1.863	2.266
Time of Administration	0.198	−0.499	2.118
Animal Sex	0.379	−3.520	8.474
Animal Type	0.315	−6.041	2.149
Dosage	0.306	−1.942	0.675
Year of Publication	0.336	−2.277	0.855
Manufacturers of MET	0.362	−0.652	1.631

#### 3.4.3 Metformin reduced TGF-β in lung tissues of pulmonary fibrosis animals

**FIGURE 6:**
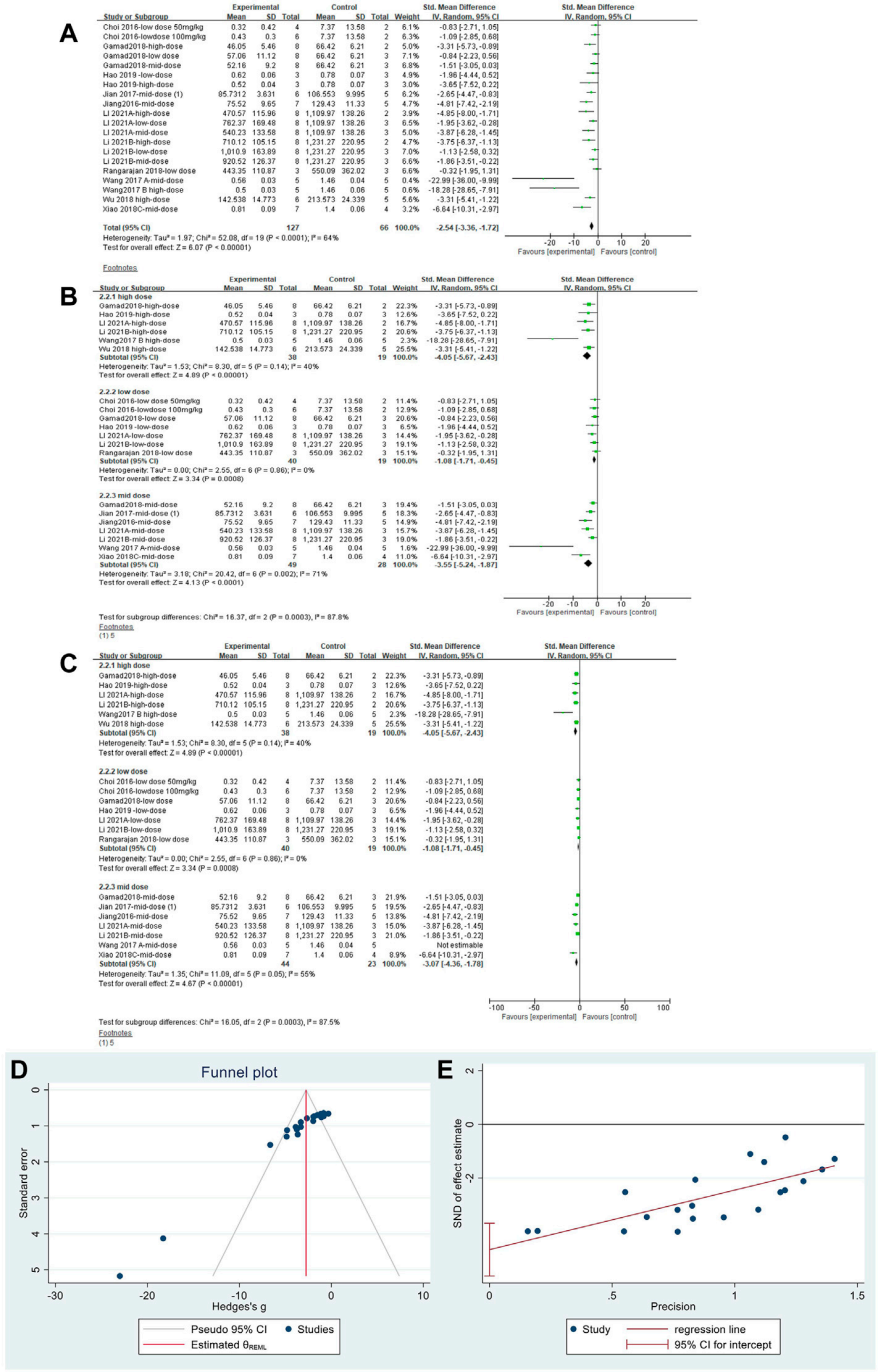
Effect of MET on TGF-β in lung tissues of PF animals. **(A)** Forest plot of TGF-β; **(B)** Forest plot of TGF-β (subgroups); **(C)** Forest plot of TGF-β [subgroups after removing Wang Y. et al., (2017)]; **(D)** Funnel plot of TGF-β; **(E)**. Egger’s test for TGF-β.

The effect sizes for TGF-β were combined in 20 independent experiments (Figure 6A), and MET was effective in reducing TGF-β levels in the PF animal models [SMD = −2.54, 95% CI (−3.36, −1.72), *p* < 0.00001]. However, heterogeneity was observed between the studies, with *I* ^*2*^ = 64%. Subgroup analysis was performed according to the different dose levels (Figure 6B). All three subgroups showed significant differences in TGF-β levels. In the high- and low-dose subgroups, heterogeneity disappeared. Heterogeneity was elevated in the mid-dose group and was mainly caused by the results from Wang Y. et al., (2017); heterogeneity was reduced after removing this literature (Figure 6C). Thus, we confirmed that the primary heterogeneity between the studies was due to the different doses of MET. The funnel plot (Figure 6D) and Egger’s test (Figure 6E) indicated no publication bias (*p* > 0.05).

#### 3.4.4 Metformin reduced hydroxyproline content in lung tissues of pulmonary fibrosis animals

**FIGURE 7:**
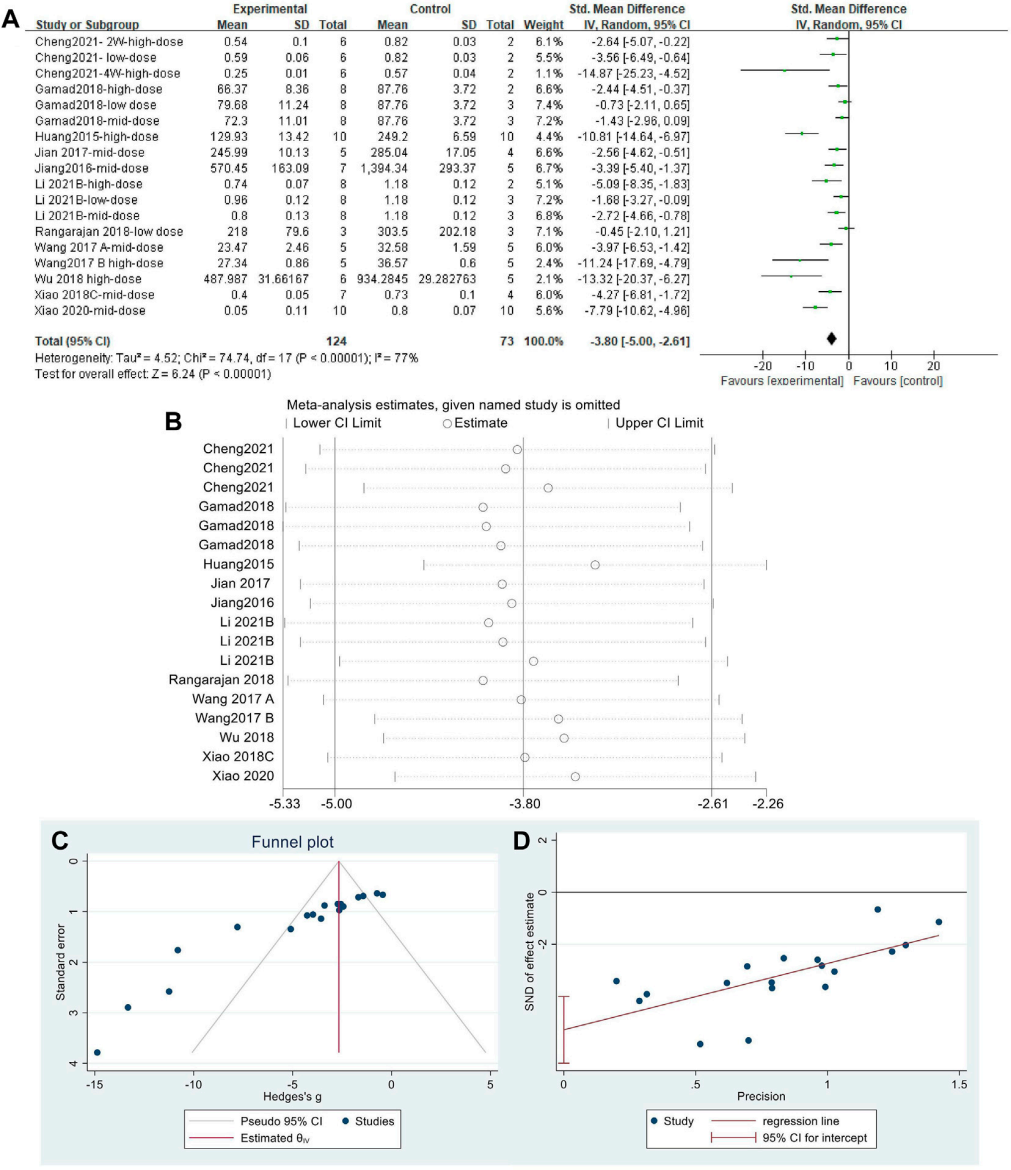
Effect of MET on HYP in lung tissues of PF animals. **(A)** Forest plot of HYP; **(B)** Sensitivity analysis of HYP; **(C)** Funnel plot of HYP; **(D)** Egger’s test for HYP.

Effect sizes were combined for HYP content of animals from 18 independent experiments (Figure 7A), which showed that MET significantly reduced HYP content in the lung tissue of PF animals compared with that in controls [SMD = −3.80, 95% CI (−5.00,−2.61), *p* < 0.00001]. However, *I* ^*2*^ = 77% indicated an inter-study heterogeneity. Sensitivity analysis was performed (Figure 7B), thereby demonstrating the stability of these findings. Publication bias was indicated based on a funnel plot (Figure 7C) and Egger’s test (Figure 7D) (*p* < 0.05).

#### 3.4.5 Metformin reduced collagen I content in lung tissues of pulmonary fibrosis animals

**FIGURE 8:**
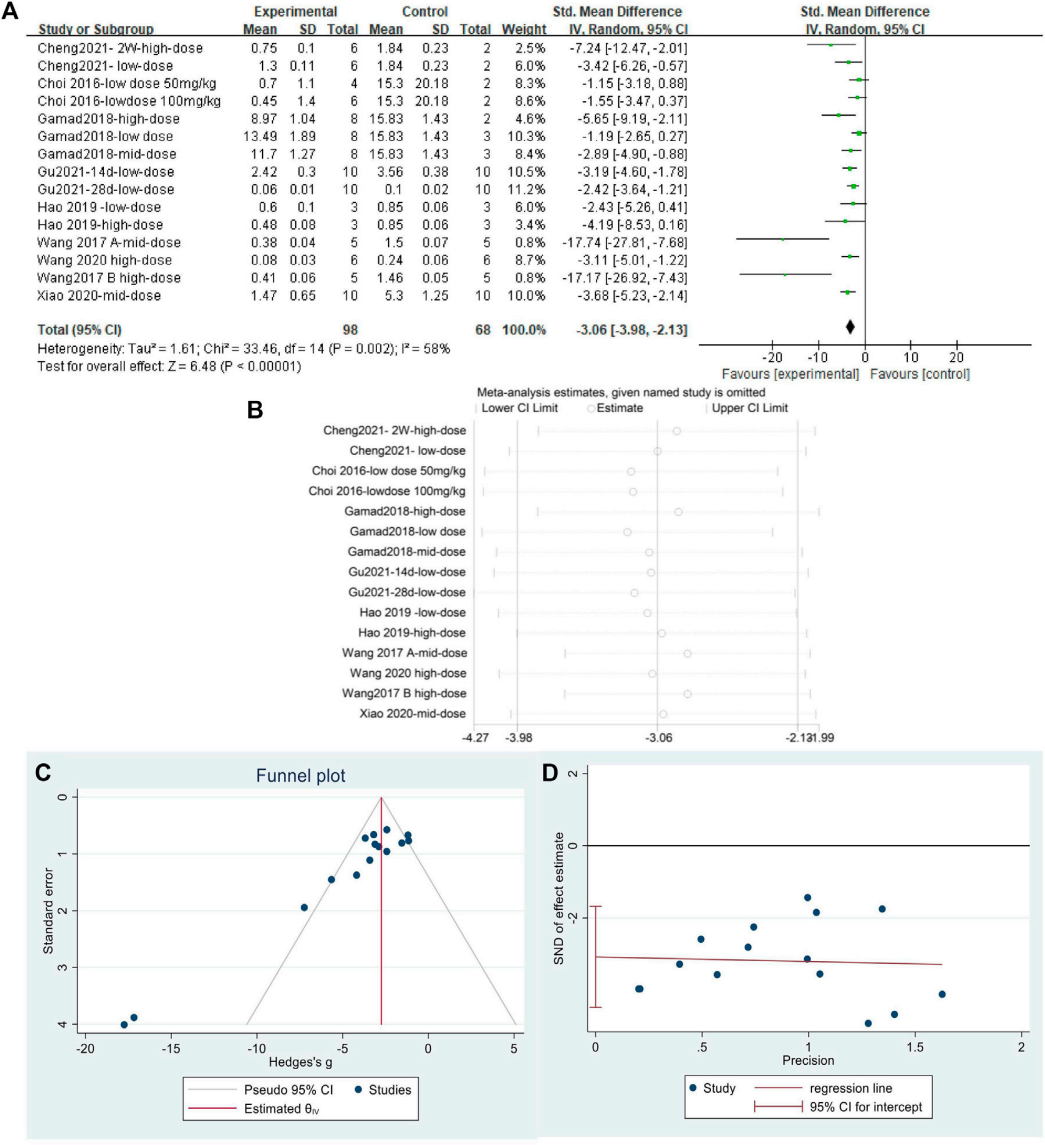
Effect of MET on collagen I in lung tissues of PF animals. **(A)** Forest plot of collagen I; **(B)** Sensitivity analysis of collagen I; **(C)** Funnel plot of collagen I; **(D)** Egger’s test for collagen I.

The effect sizes of collagen I were combined from 15 independent experiments. The results (Figure 8A) indicated that MET was effective in reducing collagen I content in the lung tissue of animal models of PF compared with that in controls [SMD = −3.06, 95% CI (−3.98, −2.13), *p* < 0.00001]. *I* ^*2*^ = 58% indicated an inter-study heterogeneity, and sensitivity analysis (Figure 8B) confirmed the stability of the findings. Publication bias was judged based on a funnel plot (Figure 8C) and Egger’s test (Figure 8D) (*p* < 0.05).

#### 3.4.6 Metformin reduced α-SMA content in lung tissues of pulmonary fibrosis animals

**FIGURE 9:**
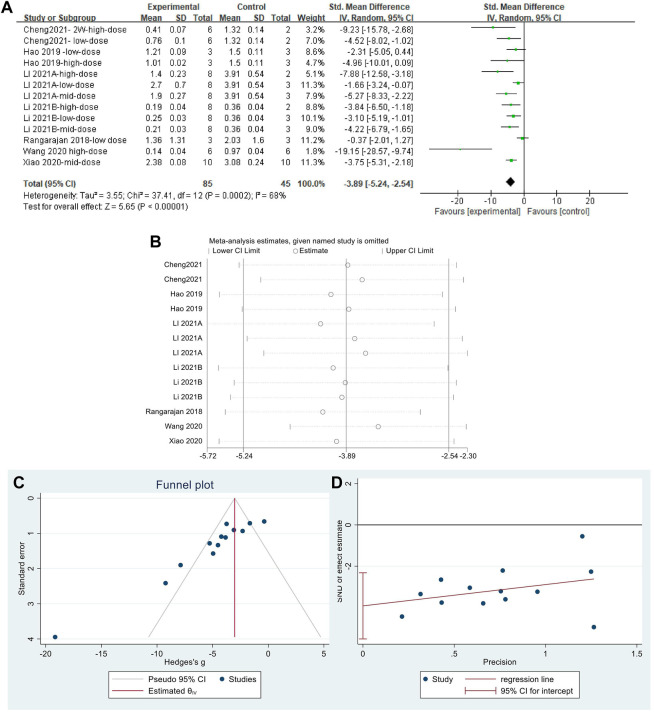
Effect of MET on α-SMA content in lung tissues of the PF animal models; **(A)** Forest plot of the α-SMA; **(B)** Sensitivity analysis of α-SMA; **(C)** Funnel plot of α-SMA; **(D)** Egger’s test for α-SMA.

The effect sizes for α-SMA were combined for 13 independent experiments (Figure 9A). MET significantly reduced α-SMA content in the lung tissue of PF animals compared with that in controls [SMD = −3.89, 95% CI (−5.24, −2.54), *p* < 0.00001]. The heterogeneity between studies was *I* ^*2*^ = 68%. Sensitivity analysis (Figure 9B) confirmed the stability of the findings with some publication bias, identified using the funnel plot (Figure 9C) and Egger’s test (Figure 9D) (*p* < 0.05).

#### 3.4.7 Metformin reduced Smad2 and Smad3 phosphorylation levels in lung tissues of pulmonary fibrosis animals with p-Smad2/3/Smad2/3

**FIGURE 10:**
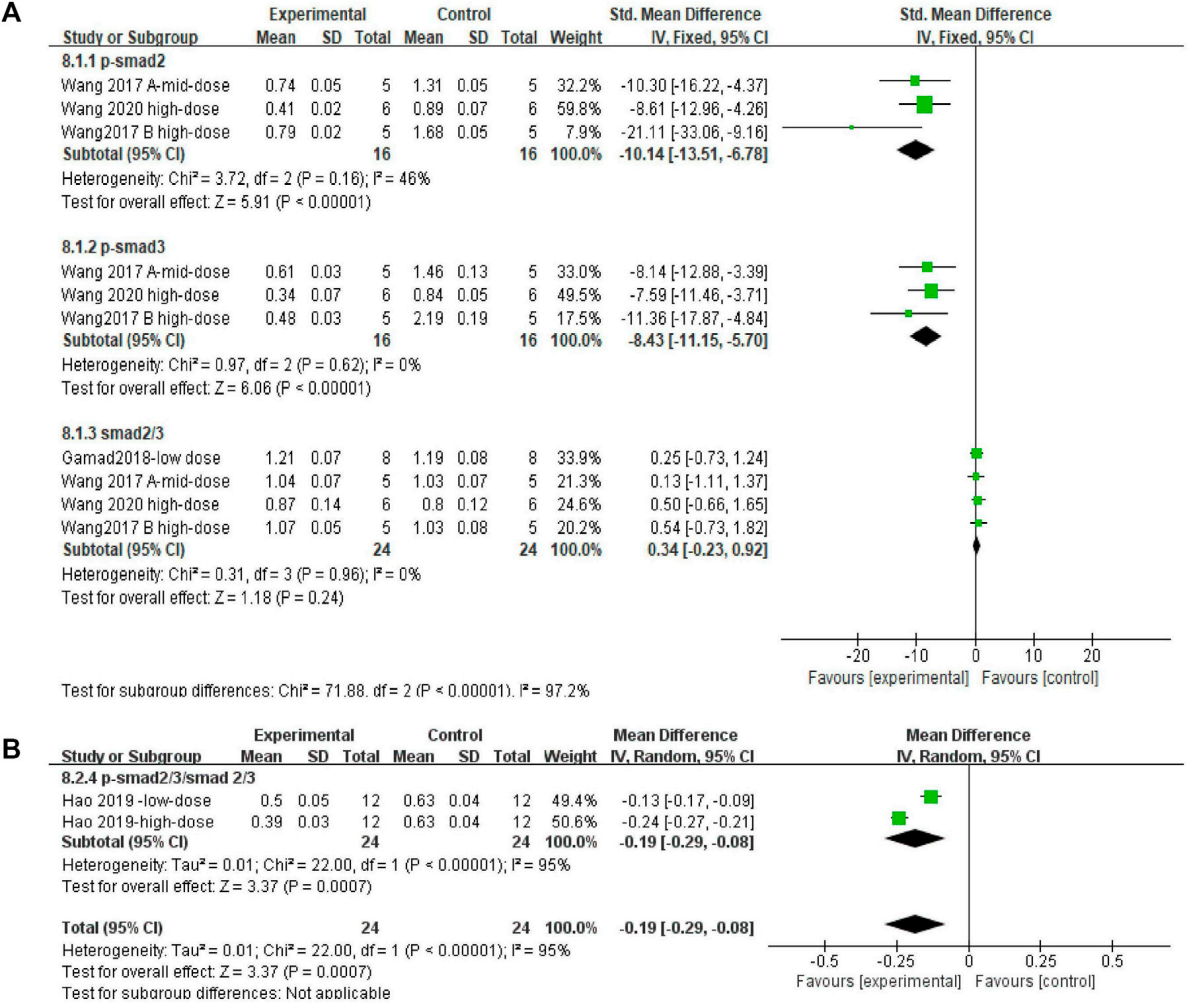
Effect of MET on Smad in lung tissues of PF animals. **(A)** Forest plot of p-Smad2, p-Smad3, and Smad2/3; **(B)** Forest plot of p-Smad2/3/Smad2/3.

Effect sizes were combined for p-Smad2, p-Smad3, Smad2/3, and p-Smad2/3/Smad2/3 in three, three, four, and two independent experiments, respectively. The Smad2/3 effect size was merged (Figures 10A and B). The use of MET significantly reduced the levels of p-Smad2 and p-Smad3 in the lung tissue of PF animals compared to those in controls (Figure 10A) [SMD = −10.14, 95% CI (−13.51, −6.78), *p* < 0.00001; SMD = −8.43, 95% CI (−11.15, −5.70), *p* < 0.00001; *I* ^*2*^ = 46% (low between-study heterogeneity; *I* ^*2*^ = 0% (no between-study heterogeneity)]. However, there was no significant difference in the change in Smad2/3 levels between the control and experimental groups [SMD = 0.34, 95% CI (−0.23, 0.92), *p* > 0.05; *I* ^*2*^ = 0% (no heterogeneity between studies)]. In the comparison of the control and experimental groups with p-Smad2/3/Smad2/3, (Figure 10B) MET significantly reduced this result [SMD = −0.19, 95% CI (−0.29, −0.08), *p* < 0.05], with heterogeneity between studies, *I* ^*2*^ = 93%.

#### 3.4.8 Metformin increased AMPK/p-AMPK content in lung tissues of pulmonary fibrosis animals

**FIGURE 11:**
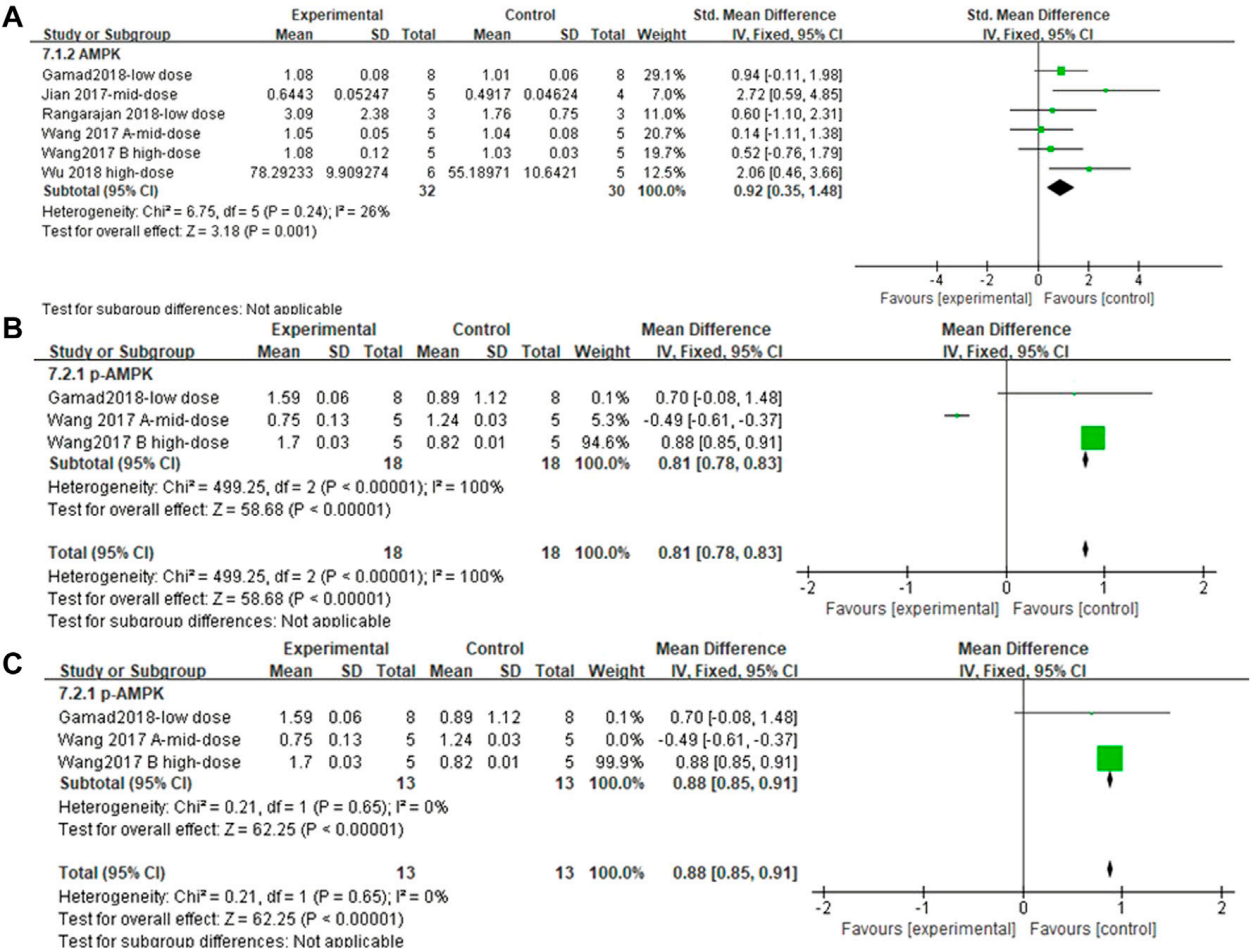
Effect of MET on AMPK/p-AMPK. **(A)** Forest plot of AMPK; **(B)** Forest plot of p-AMPK; **(C)** Forest plot of p-AMPK [after removing Wang Y. et al., (2017)].

Five and three independent experiments were combined to determine the lung tissue AMPK and p-AMPK effect sizes, respectively. The results showed (Figures 11A and B) that the treatment of PF animals with MET significantly reduced AMPK and p-AMPK levels [SMD = 0.92, 95% CI (0.35, 1.48), *p* < 0.001; SMD = 0.81, 95% CI (0.78, 0.83), *p* < 0.001]. There was no heterogeneity in the effect sizes for AMPK; *p* > 0.24, *I* ^*2*^ = 26%. The combined effect sizes of p-AMPK showed heterogeneity between studies and reduced after sensitivity analysis by removing the results from Wang Y. et al., (2017) (Figure 11C).

#### 3.4.9 Metformin significantly reduced ERK1/2 phosphorylation levels in lung tissues of pulmonary fibrosis animals

**FIGURE 12:**
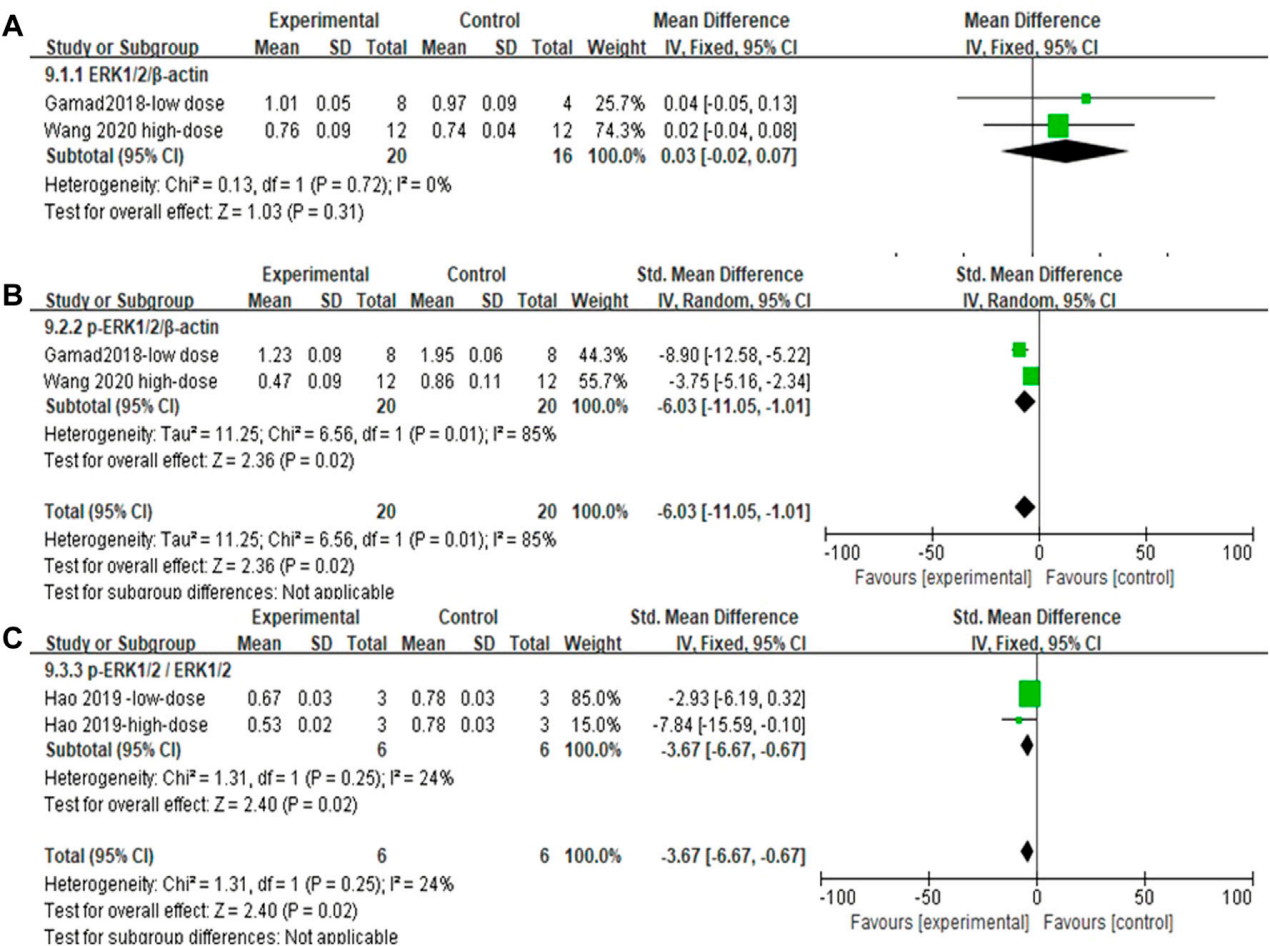
Effect of MET on ERK1/2 phosphorylation levels in lung tissues of PF animals. **(A)** Forest plot of ERK1/2; **(B)** Forest plot of ERK1/2p-ERK1/2; **(C)** Forest plot of ERK1/2p-ERK1/2/ERK1/2.

The effect sizes of ERK1/2, p-ERK1/2, and p-ERK1/2/ERK1/2 (each metric contained two independent experiments) in the lung tissue of PF animals were combined for the included studies (Figures 12A–C). There was no significant difference in ERK1/2/β-actin levels after MET treatment compared to those in controls [SMD = 0.03, 95% CI (−0.02, 0.07), *p* > 0.05]. In the experimental group, the use of MET significantly reduced the phosphorylation levels of both p-ERK1/2/β-actin and p-ERK1/2/ERK1/2/β-actin overall [SMD = −6.03, 95% CI (−11.05, −1.01), *p* < 0.05; SMD = −3.67, 95% CI (−6.67, −0.67), *p* < 0.05] compared to those in the control group (Figures 12B and C). *I* ^*2*^ = 0% (with no heterogeneity between studies), *I* ^*2*^ = 85% (with high heterogeneity between studies), *I* ^*2*^ = 24% (with no heterogeneity between studies) are the results of sensitivity analyses for ERK1/2, p-ERK1/2, and p-ERK1/2/ERK1/2, respectively.

#### 3.4.10 Metformin reduced mortality in pulmonary fibrosis animals

**FIGURE 13:**
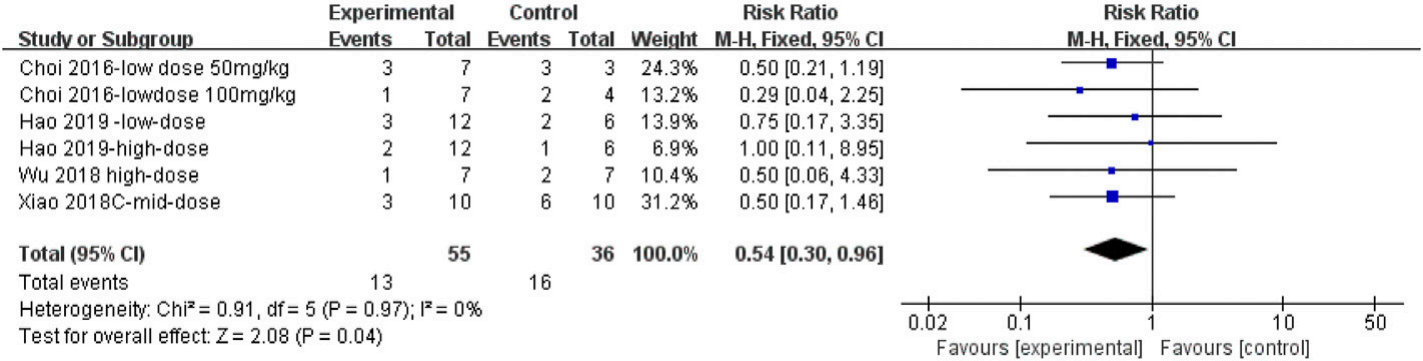
Effect of MET on mortality in animals.

The effect sizes for mortality in PF animal models were combined for six independent experiments (Figure 13), and the protective effect of MET on mortality was significant compared to that of controls [RR = 0.54, 95% CI (0.30, 0.96), *p* < 0.05]. *I* ^*2*^ = 0%, indicating no heterogeneity between studies.

## 4 Discussion

The non-standard use of a drug is a method to improve treatment and understanding of the disease. Compared to traditional drug development, exploring the treatment scope of existing drugs is more convenient, cheaper, and less risky (Jin and Wong, 2014; Tzouvelekis et al., 2018). A new function of the old drug MET, the anti-fibrotic effect, has been explored. Clinical studies using MET in treating patients with IPF are currently scarce, with two previous studies indicating no significant effect of MET on the clinical outcomes in patients with PF (Spagnolo et al., 2018; Lambert et al., 2021). However, both studies were limited by their small sample sizes (*n* = 28 and *n* = 71). In contrast, a recent retrospective cohort study that included 3,599 patients with IPF and T2DM showed a significant 54% reduction in all-cause mortality with MET treatment (Teague et al., 2022). This effect was substantially better than that of the current anti-fibrotic agents (23%) (Dempsey et al., 2019). This study confirms that our proposal for the use of MET for the treatment of PF is both scientific and innovative and provides strong practical evidence for our research. However, it is worth noting that this study observed the effect of MET in patients with PF and T2DM, and there is insufficient evidence of a direct causal relationship between MET and PF. In addition to its anti-fibrotic effect, MET also has cardioprotective and hypoglycemic effects (Holman et al., 2008; Roumie et al., 2012), which may be responsible for reducing mortality in patients with PF and T2DM (Teague et al., 2022). Therefore, further clinical trials are required. Preclinical studies often support the development of clinical trials. There are many preclinical studies on MET for PF animal models, but reviews summarizing these studies are scarce or incomplete. In addition, some of the published reviews on the mechanisms of MET for PF lack relatively complete experimental evidence or focus on specific aspects of the anti-PF mechanisms of MET. Systematic evaluation based on evidence is conducive to improving the quality of animal experiments and transforming from preclinical to clinical research. 

### 4.1 Summary of current findings

To the best of our knowledge, this is the first systematic review and meta-analysis of the effects of MET in animal models of PF. During the literature screening process, we initially selected 20 articles but eventually included only 19 articles after careful reading. An article (Kheirollahi et al., 2019) was excluded from this study because it did not quantify the scoring of inflammation, degree of fibrosis, or collagen content in Masson-stained sections of animal lung tissue. Although the article used the degree of fibrosis (%) for evaluation, it was not the desired outcome indicator. A total of 19 studies that included 29 separate experiments involving 368 animals in both experimental and control groups were eventually used in this study. This meta-analysis showed that MET significantly reduced lung inflammation, fibrosis, and its associated markers in PF animal models. MET administration directly or indirectly improved almost all observed markers. The results of this study suggest that MET is a promising candidate for the treatment and suppression of PF.

### 4.2 Heterogeneity

Due to the different experimental designs included in the studies, there were multiple variables, such as modeling method, route of administration, time of administration, animal sex, animal type, dosage, year of publication, and manufacturer of MET, which may have contributed to the high heterogeneity of some of the outcomes (Bailoo et al., 2014; Chaudhari et al., 2020). We anticipated high heterogeneity before conducting the meta-analysis. Each independent experiment clearly stated the dose of MET and the dose fluctuation range in the included studies was 50–500 mg/kg. Therefore, the dose types in the included studies were classified according to the dose classification method mentioned in the Materials and Methods Section 2.5. Subgroups were introduced to calculate the fibrosis score and TGF-β level because their heterogeneity was too high (Lin et al., 2020). These two results demonstrated that different doses of MET could produce the same effect. Subgroup analysis combined with meta-regression results showed that dose did not affect the fibrosis score. There was no heterogeneity between the studies in the calculation of the inflammation score. Therefore, it cannot be determined whether the dose significantly affected the heterogeneity of the inflammation score. Moreover, regarding TGF-β, it has been proven that the dose is the main factor affecting the level of heterogeneity because the heterogeneity reduced to varying degrees after subgroup analysis. Therefore, based on the existing evidence, there is no significant effect between dosage and the heterogeneity of fibrosis or inflammation score; however, the heterogeneity reduced in TGF-β analysis. In the fibrosis score analysis after subgroup analysis, dose and factors other than it were considered. The fibrosis score and the dose administered again proved to be unrelated in the regression analysis. We explored the influence of other possible factors on heterogeneity between studies. We conducted a meta-regression analysis of eight factors, including the mode of modeling, route of administration, time of administration, sex of animals, type of animals, the dosage of administration, year of publication, and MET manufacturer. However, these variables did not significantly affect heterogeneity. We believe that some other factors may contribute to high heterogeneity, such as subjective conditions, differences in scores given by different investigators, experimental conditions, and differences in testing equipment.

### 4.3 Interpretation and discussion of the study results

#### 4.3.1 Selection of animal types

This study did not limit the types of experimental animals, as several animals have been used to construct animal models of PF (Tashiro et al., 2017). Different species of PF animal models are used to reflect patients with PF, a disease difficult to replicate fully. The animals used in the included studies were all male and female murine (mice and rats), which are commonly used for PF modeling. Redente et al. argued that male animals respond better to PF than females (Redente et al., 2011). However, in the present study, there was no significant effect of gender on the findings based on the meta-regression of fibrosis scores (*p* > 0.05). It has been suggested that fibrosis occurring in young mice after receiving a single dose of bleomycin spontaneously regresses, but this was not observed in older mice (Degryse et al., 2010). Multiple modeling injections into young mice can better characterize human fibrosis (Degryse and Lawson, 2011; Wang et al., 2019). Of the included studies that used bleomycin interventions, only one mentioned modeling with multiple doses of bleomycin; the rest used a single dose for modeling. Of the included studies, only two mentioned the age of the animals. This suggests that in future relevant experiments, researchers should try to use older rats or administer multiple doses to younger rats to optimize the animal model and demonstrate the characteristics of human IPF (Degryse et al., 2010).

#### 4.3.2 Modeling method

The current meta-analysis did not restrict the mode of modeling to better demonstrate the various states and etiologies of the PF development process. Bleomycin was used for modeling in 63% of the included studies (12), which aligns with the current consensus for studying animal models of PF (Jenkins et al., 2017). However, animal models of bleomycin are problematic, as previously described. Fibrosis in young mice may regress spontaneously (Jenkins et al., 2017) and is often bleomycin-induced. Bleomycin-induced PF is not fully representative of IPF because of the acute lung tissue damage that often leads to high mortality in animals (Tashiro et al., 2017). Another 16% 3) of the included studies used silica interventions, and 21% 4) used radiation interventions. Silica-induced PF demonstrated silicosis-induced PF (Davis et al., 1998), and the resulting PF lesions tended to be more persistent (Barbarin et al., 2004; Tashiro et al., 2017). Radiation-induced PF tends to be a slow process and is less dependent on TGF-β (Haston, 2012). The inclusion of these two modeling studies can, to some extent, compensate for the limitations of the bleomycin-induced PF model and provide a more comprehensive response to the therapeutic effect of MET on PF.

#### 4.3.3 The effect and mechanism of metformin on animal models of pulmonary fibrosis

In this meta-analysis, the effect of MET on PF was divided into two aspects. MET administration reduced lung inflammation scores, and acute inflammation-dominated lung injury was the main feature of animal PF models in the early stages of PF development (1–10 days) (Moeller et al., 2008; Tashiro et al., 2017). Infiltration of inflammatory cells and damage to alveolar epithelial cells were the main manifestations during this period. This meta-analysis demonstrated that MET had a significant effect on the inflammatory state of the lungs at this stage. On the other hand, fibrosis in the lung generally starts with the deposition of the ECM. The progression of fibrosis on days 10–21, especially on day 14, was the peak of development (Tashiro et al., 2017). The present meta-analysis demonstrated the effect of MET on the fibrosis stage with regard to the fibrosis score of pathological lung tissue and the expression of fibrosis factors HYP, collagen I, and α-SMA. The use of MET significantly improves alveolar wall thickness and reduces collagen deposition in lung tissues, thereby reducing the extent of fibrosis in the lung tissue (Hao et al., 2019).

In addition, we explored the underlying mechanisms of MET in PF amelioration. The essence of PF is the excessive deposition of collagen and ECM proteins (Jones et al., 2018). The reasons behind considering collagen I, HYP, and α-SMA as factors to monitor the extent of fibrosis were: 1) Collagen I, the primary collagen involved in the ECM, is used as a targeting probe (Désogère et al., 2017) to determine the extent of fibrosis. HYP is a major component of collagen and is often used as an indicator of the total collagen content (Zhao et al., 2015). Investigators recommend HYP content reduction as the primary criterion for evaluating anti-fibrotic efficacy in preclinical animal models (Jenkins et al., 2017). α-SMA may reflect the levels of activated fibroblasts (Li et al., 2011). This study demonstrates the improvement in the degree of fibrosis (collagen I), anti-fibrotic effect (HYP), and level of collagen fibril activation (α-SMA) during MET treatment. Ultimately, MET reduced these three indicators, indicating that MET can influence fibroblast activation by modulating α-SMA, which improves the degree of fibrosis in the lung by reducing collagen I levels and exerting an anti-fibrotic effect. 2) Collagen I is a protein, and HYP is an amino acid (Xu et al., 2021). Taking these two markers as the research results is convenient for reactions at different functional levels. 3) In lung tissues, collagen I and III are the most important collagen proteins, and HYP reflects the overall collagen deposition. Selecting HYP and collagen I is also conducive to further exploring the mechanism and pathway of collagen deposition and determining whether MET improves PF by reducing the collagen I pathway. MET acts mainly through the regulation of AMPK (Rangarajan et al., 2018). It is believed that the activation of AMPK can inhibit the most crucial part of the PF process, the TGF-β signaling pathway (Wu et al., 2021). Therefore, this meta-analysis explored several indicators related to TGF-β, including TGF-β, AMPK, Smad, and ERK. The results showed that MET significantly affected AMPK and p-AMPK levels and significantly elevated TGF-β, Smad (p-Smad2, p-Smad3, and p-Smad2/3), and ERK (p-ERK1/2/β-actin and p-ERK1/2/ERK1/2) levels. This suggests the ability of MET to enhance anti-fibrotic and lung-protective effects by activating AMPK in animals post MET administration (Wynn and Ramalingam, 2012). This meta-analysis also demonstrated that MET inhibited the effect of EMT, as represented by α-SMA, which further inhibited the occurrence of PF. Notably, in the final merging of effect sizes for p-AMPK, we removed Wang Y. et al., (2017) because of the high heterogeneity of p-AMPK. MET did not result in elevated AMPK phosphorylation. This validates a previous view that MET may have non-AMPK–dependent pathways to prevent PF (Feng et al., 2017). In addition, not all indicators in this meta-analysis yielded satisfactory results. The effect of MET on Smad2/3 and ERK1/2/β-actin was *p* > 0.05, i.e., there was no significant difference between the control and experimental groups. However, the comparative results of the effect sizes associated with p-Smad2, p-Smad3, p-Smad2/3, p-ERK1/2/β-actin, and p-ERK1/2/ERK1/2 were *p* < 0.05, which was in accordance with our expectations. We speculated that the main reason for this discrepancy was that the number of included studies reporting the two outcomes of Smad2/3 and ERK1/2/β-actin was low, resulting in a combined effect size that does not reflect a broader and more realistic picture. However, it is possible that the time point selection of the assay resulted in a small difference between these two outcomes in the comparison of experimental and control groups and did not prove their significance when combining the effect sizes.

**FIGURE 14:**
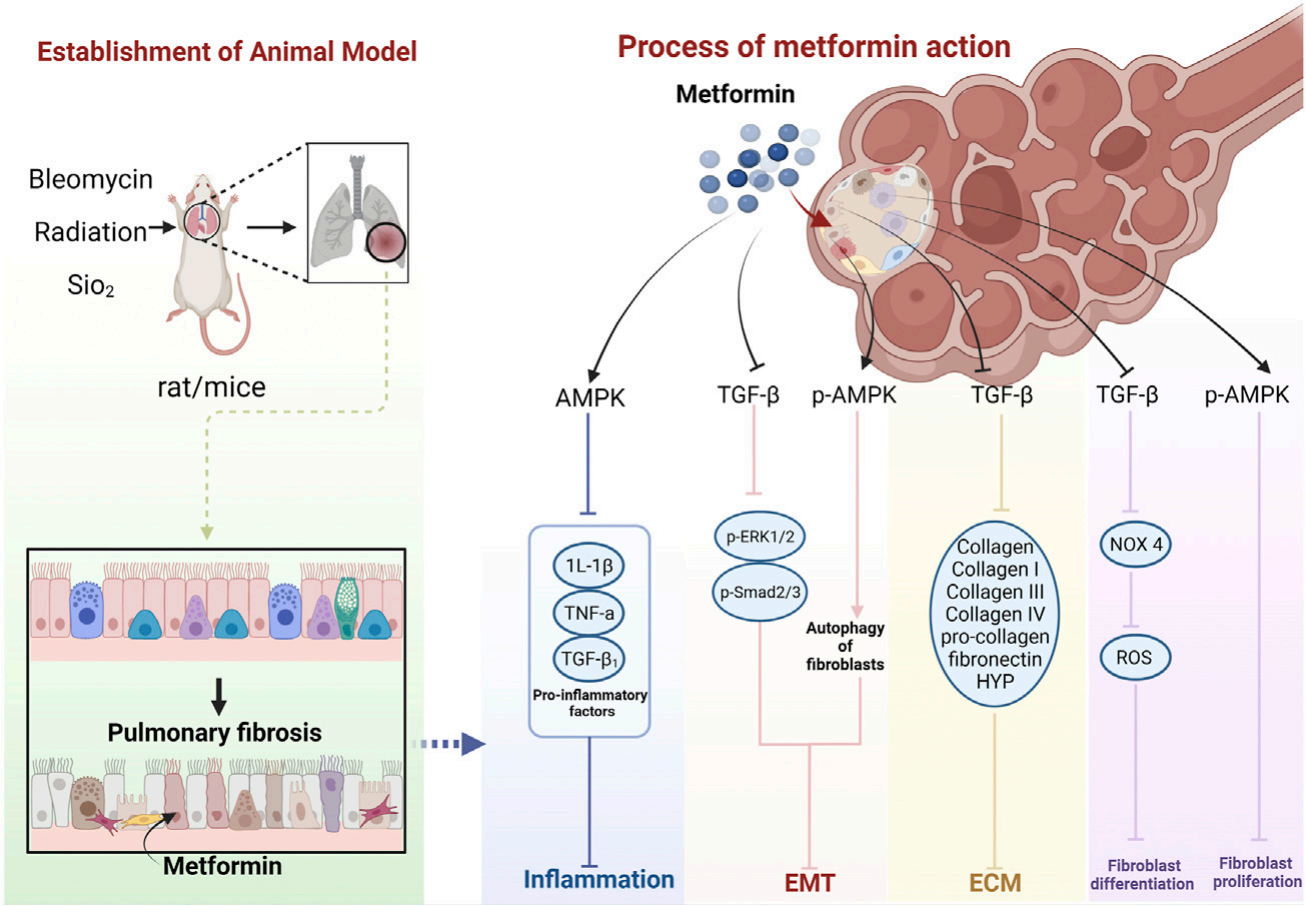
Schematic representation of the possible molecular mechanism of MET inhibition of PF. (Created using BioRender.com).

The cellular and molecular mechanisms underlying MET treatment in PF are unknown. The current focus is mainly on AMPK, suggesting that MET inhibits and ameliorates PF mainly through the AMPK pathway. However, there may also be non-AMPK pathway–dependent mechanisms (Wang Y. et al., 2017) (Figure 14)

##### 4.3.3.1 Inhibitory effect of metformin on inflammatory response

MET further inhibits the inflammatory response by activating AMPK and inhibiting pro-inflammatory factors, such as IL-1β, TNF-α, and TGF-β1 (Jian, 2017; Li et al., 2021a; Li et al., 2021b).

##### 4.3.3.2 Inhibition of EMT by metformin

MET inhibits the TGF-β pathway in lung fibroblasts, thereby diminishing the activity of its downstream molecules p-Smad2, p-Smad3, and p-ERK1/2 proteins (Wang et al., 2020), and inhibition of Smad2/3 phosphorylation can enhance E-Cad transcription (Huang, 2015), while leading to decreased expression of proteins, such as α-SMA (Li et al., 2021a; Li et al., 2021b), ultimately inhibiting EMT. MET can also inhibit EMT by upregulating p-AMPK and activating autophagy (Li et al., 2021a).

##### 4.3.3.3 Inhibitory effect of metformin on fibroblast differentiation and proliferation

MET activates AMPK phosphorylation and inhibits fibroblast proliferation. AMPK can inhibit NADPH oxidase 4 (NOX4) expression downstream of TGF-β and reduce ROS production, thus, inhibiting Smad phosphorylation and myofibroblast differentiation (Sato et al., 2016).

##### 4.3.3.4 Metformin reduces extracellular matrix deposition

MET downregulates TGF-β expression, which, in turn, inhibits COL, COL-I, COL III, COL-IV, pro-COL, and fibronectin production (Choi et al., 2016; Wang J. et al., 2017; Gamad et al., 2018; Hao et al., 2019), leading to a decrease in ECM deposition. HYP is a product of collagen catabolism, and an increase in its level implies an increased expression of collagen in tissues. This suggests that MET can reduce collagen in lung tissues by inhibiting HYP production, thus, reducing ECM deposition (Jian, 2017). MET also decreases TGF-β1, Smad2, and Smad3 phosphorylation levels, and the process of collagen I inhibition can be affected through a non-AMPK–dependent pathway (Wang Y. et al., 2017). In addition, MET may produce anti-fibrotic effects through other non-AMPK pathways (Gao et al., 2020). MET exerts an anti-fibrotic effect by inhibiting insulin-like growth factor 1 (IGF-1) (Xiao et al., 2020). The MET hypoglycemic mechanism of MET also functions through IGF-1. Inhibition of IGF-1 prevents the activation of IGF-1 receptors and inhibits IGF-1-mediated downregulation of insulin sensitivity (Kang et al., 2016). MET can also exert anti-PF effects by inhibiting the regulation of NOX4 (Sato et al., 2016). Similarly, MET has been shown to prevent and treat T2DM and its complications, such as diabetic cataracts, by inhibiting NADPH oxidase and preventing ROS accumulation (Chen et al., 2022). MET may also induce lipid differentiation through activation of BMP2 release and the PPARγ phosphorylation pathway (Kheirollahi et al., 2019), a pathway that has also been reported to regulate glycemic effects (Schultze et al., 2012). It can also inhibit transglutaminase 2 (TG2) upstream of TGF-β1, which is involved in inhibiting the PI3K-Akt, TGF-β/Smad, and RTK/RAS/ERK pathways to exhibit an anti-fibrosis role (Hsu et al., 2017; Wang et al., 2020). Interestingly, the hypoglycemic effect of MET is also mediated by the PI3K-Akt pathway but with positive activation (Schultze et al., 2012).

In summary, the effects of MET on animal models of PF may proceed through four aspects: inhibition of the body’s oxidative stress and inflammatory response by the AMPK pathway, EMT, fibroblast proliferation and differentiation, and ECM deposition; however, they can also function through non-AMPK–dependent pathways.

### 4.4 Limitations

Although this study’s careful and detailed analysis was based on the Handbook for Systematic Evaluation of Animal Intervention Studies (de Vries et al., 2015), the studies included in this meta-analysis have some limitations. First, animal models cannot fully replicate the full range of human diseases (Perel et al., 2007). Furthermore, rodents recovered much faster than humans did. Therefore, we cannot confirm whether the results of the current meta-analysis can be applied to PF treatment in humans. Second, the age of the animals was poorly described by the investigators in the included studies, with only two studies mentioning the age and sex of the animals. Because rodent age and sex affect the effect of MET (Chaudhari et al., 2020), we cannot affirm whether the animal models in the current included studies are fully representative of the effect of MET on the primary onset population of PF. In addition, the included studies lacked many basic methods to reduce bias, such as concealment allocation and blinding of animal-keepers and investigators. Better quality animal models of PF can translate to human patients with PF and predict the effects of drugs in humans in a better way (Perel et al., 2007). Future research should focus on improving experimental methods, modeling approaches, and the quality of reporting of animal experiments to provide high-quality evidence.

### 4.5 Future of metformin for pulmonary fibrosis treatment

Research on the mechanism of MET in the treatment of PF requires further preclinical studies. In addition, the following issues may need to be addressed when transitioning from preclinical to clinical studies. 1) There may be a drug dose conversion problem with MET use in patients with PF. MET is a therapeutic agent for the treatment of systemic diseases, whereas PF is a restrictive disease. If the effect of MET on PF is to be studied, efficient drug delivery to the lungs must be considered to optimize the drug concentration in the lungs (Manning et al., 2019). To date, the preclinical studies used large doses of MET compared with regular doses to enable adequate efficacy in the lungs of animals. 2) Biomarkers in patients with IPF are often specific (Guiot et al., 2017), affecting patient mortality and drug response. Therefore, the clinical effectiveness of MET in patients with PF should be combined with ongoing studies on PF-specific biomarkers (Raghu et al., 2018). 3) PF may not be clinically isolated, often existing in combination with other diseases, and may be influenced by factors such as smoking, age, and sex of the patient, suggesting that confounding factors often influence these studies (Bai et al., 2021). This may be the reason for some bias in the studies and the impact of the results. Nevertheless, we hope that clinical studies on MET for PF will be conducted in the future. This is because there is more than adequate evidence from preclinical studies and positive evidence from retrospective cohort studies with larger sample sizes. However, given the respect and protection of life, future investigators must address the possible abovementioned shortcomings. 

## 5 Conclusion

This study is the first systematic review and meta-analysis of the effects of MET in animal models of PF. The present meta-analysis showed that MET significantly improved PF in animal models. MET may reverse and attenuate PF by activating AMPK and inhibiting TGF-β, α-SMA, and other pathways, ultimately inhibiting the phenotypes of fibroblast proliferation and differentiation, EMT, collagen deposition, and inflammatory response in lung tissues. In conclusion, this study suggests that MET may be a potential candidate for the treatment of PF.

## Data Availability

The original contributions presented in the study are included in the article/Supplementary Materials; further inquiries can be directed to the corresponding authors.
