# Supplementary material for: Effectiveness and mechanism of metformin in animal models of pulmonary fibrosis: A preclinical systematic review and meta-analysis
Source: Front Pharmacol. 2022 Sep 6;13:948101. doi: 10.3389/fphar.2022.948101 (PMC9485720; doi:10.3389/fphar.2022.948101)
Supplement: Supplementary file 1 [file DataSheet1.docx]

Supplementary Material

[Supplementary Table S1 Search strategy 2](#_Toc110778467)

[Supplementary File S1 The PRISMA checklist of this meta-analysis 9](#_Toc110778468)

[Supplementary Table S2 Basic characteristics of the included studies 13](#_Toc110778469)

[Supplementary Table S3 Modeling and Drug Administration in the Included Studies 15](#_Toc110778470)

## Supplementary Table S1 Search strategy

| **Databases** | **Search items** | **Number** |
| --- | --- | --- |
| **Pubmed** | #1 Metformin  #2 Dimethylbiguanidine  #3 Dimethylguanylguanidine  #4 Glucophage  #5 Metformin Hydrochloride  #6 Hydrochloride, Metformin  #7 Metformin HCl  #8 HCl, Metformin  #9 OR/#1-#8  #10 Pulmonary Fibrosis  #11 Fibrosis, Pulmonary  #12 Pulmonary Fibroses  #13 Fibroses, Pulmonary  #14 Alveolitis, Fibrosing  #15 Alveolitides, Fibrosing  #16 Fibrosing Alveolitides  #17 Fibrosing Alveolitis  #18 Idiopathic Diffuse Interstitial Pulmonary Fibrosis  #19 Idiopathic Pulmonary Fibrosis  #20 Pulmonary Fibroses, Idiopathic  #21 Idiopathic Fibrosing Alveolitis, Chronic Form  #22 Fibrosing Alveolitis, Cryptogenic  #23 Fibrocystic Pulmonary Dysplasia  #24 Dysplasia, Fibrocystic Pulmonary  #25 Fibrocystic Pulmonary Dysplasias  #26 Pulmonary Dysplasia, Fibrocystic  #27 Cryptogenic Fibrosing Alveolitis  #28 Cryptogenic Fibrosing Alveolitides  #29 Fibrosing Alveolitides, Cryptogenic  #30 Pulmonary Fibrosis, Idiopathic  #31 Usual Interstitial Pneumonia  #32 Interstitial Pneumonia, Usual  #33 Usual Interstitial Pneumonias  #34 Interstitial Pneumonitis, Usual  #35 Pneumonitides, Usual Interstitial  #36 Pneumonitis, Usual Interstitial  #37 Usual Interstitial Pneumonitides  #38 Usual Interstitial Pneumonitis  #39 Familial Idiopathic Pulmonary Fibrosis  #40 Idiopathic Pulmonary Fibrosis, Familial  #41 OR/#10-#40  #42 Models, Animal  #43 Animal Model  #44 Animal Models  #45 Model, Animal  #46 Laboratory Animal Models  #47 Animal Model, Laboratory  #48 Animal Models, Laboratory  #49 Laboratory Animal Model  #50 Model, Laboratory Animal  #51 Models, Laboratory Anima  #52 Experimental Animal Models  #53 Animal Model, Experimental  #54 Animal Models, Experimental  #55 Experimental Animal Model  #56 Model, Experimental Anima  #57 Models, Experimental Animal  #58 OR/#42-#57  #59 #9 AND #41 AND #58 | 7 |
| **Cochrane**  **Library** | #1 MeSH descriptor: [Metformin] explode all trees  #2 (Dimethylbiguanidine):ti,ab,kw OR (Dimethylguanidine):ti,ab,kw OR (Glucophage):ti,ab,kw OR (Metformin Hydrochloride):ti,ab,kw OR (Hydrochloride, Metformin):ti,ab,kw OR (Metformin, HCI) :ti,ab,kw OR (HCI, Metformin):ti,ab,kw (Word variations have been searched)  #3 #1 OR #2  #4 MeSH descriptor: [Pulmonary Fibrosis] explode all trees  #5 (lung fibrosis):ti,ab,kw OR (Fibrosis, Pulmonary):ti,ab,kw OR (Pulmonary Fibroses):ti,ab,kw OR (Fibroses, Pulmonary):ti,ab,kw OR (Alveolitis, Fibrosing):ti,ab,kw OR (Alveolitides, Fibrosing):ti,ab,kw OR (Fibrosing Alveolitides):ti,ab,kw OR (Fibrosing Alveolitis):ti,ab,kw OR (Idiopathic Diffuse Interstital Pulmonary Fibrosis):ti,ab,kw (Word variations have been searched)  #6 #4 OR #5  #7 MeSH descriptor: [Models, Animal] explode all trees  #8 (Animal Model):ti,ab,kw OR (Animal Models):ti,ab,kw OR (Model, Animal):ti,ab,kw OR (Laboratory Animal Models):ti,ab,kw OR (Animal Model, Laboratory):ti,ab,kw OR (Animal Models, Laboratory):ti,ab,kw OR (Laboratory Animal Model):ti,ab,kw OR (Model, Laboratory Animal):ti,ab,kw OR (Models, Laboratory Animal):ti,ab,kw OR (Experimental Animal Models):ti,ab,kw OR (Animal Model, Experimental):ti,ab,kw OR (Animal Models, Experimental):ti,ab,kw OR (Model, Experimental Animal):ti,ab,kw OR (Models, Experimental Animal):ti,ab,kw OR (Experimental Animal Model):ti,ab,kw (Word variations have been searched)  #9 #7 OR #8  #10 MeSH descriptor: [Idiopathic Pulmonray Fibrosis] explode all trees  #11 (Pulmonray Fibrosis, Idiopathic):ti,ab,kw OR (Idiopathic Fibrosis Alveolitis, Chronic Form):ti,ab,kw OR (Fibrosing Alveolitis, Cryptogenic):ti,ab,kw OR (Fibrocystic Pulmonary Dysplasia):ti,ab,kw OR (Dysplasia, Fibrocystic Pulmonary):ti,ab,kw OR (Fibrocystic Pulmonary Dysplasias):ti,ab,kw OR (Pulmonary Dysplasia, Fibrocystic):ti,ab,kw OR (Cryptogenic Fibrosing Alveolitis):ti,ab,kw OR (Cryptogenic Fibrosing Alveolitides):ti,ab,kw OR (Fibrosing Alveolitides, Cryptogenic):ti,ab,kw OR (Pulmonray Fibrosis, Idiopathic):ti,ab,kw OR (Usual Interstitial Pneumonia):ti,ab,kw OR (Interstitial Pneumonia, Usual):ti,ab,kw OR (Usual Interstitial Pneumonias):ti,ab,kw OR (Interstitial Pneumonias, Usual):ti,ab,kw OR (Pneumonitides, Usual Interstitial):ti,ab,kw OR (Pneumonitis, Usual Interstitial):ti,ab,kw OR (Usual Interstitial Pneumonitides):ti,ab,kw OR (Usual Interstitial Pneumonitis):ti,ab,kw OR (Familial Idiopathnic Pulmonary Fibrosis):ti,ab,kw OR (Idiopathnic Pulmonary Fibrosis, Familial) :ti,ab,kw (Word variations have been searched)  #12 #10 OR #11  #13 #12 OR #6  #14 #3 AND #13 AND #9 | 0 |
| **EMBASE** | #1 metformin  #2 dimethylbiguanidine  #3 dimethylguanylguanidine  #4 glucophage  #5 metformin hydrochloride  #6 hydrochloride, metformin  #7 metformin hcl  #8 hcl, metformin  #9 OR/#1-#8  #10 animal model  #11 models, animal  #12 animal models  #13 model, animal  #14 laboratory animal models  #15 animal model, laboratory  #16 animal models, laboratory  #17 laboratory animal model  #18 model, laboratory animal  #19 models, laboratory animal  #20 experimental animal models  #21 animal model, experimental  #22 animal models, experimental  #23 experimental animal model  #24 model, experimental animal  #25 models, experimental animal  #26 OR/#10-#25  #27 lung fibrosis  #28 fibrosis, pulmonary  #29 pulmonary fibroses  #30 fibroses, pulmonary  #31 alveolitis, fibrosing  #32 alveolitides, fibrosing  #33 fibrosing alveolitides  #34 fibrosing alveolitis  #35 idiopathic diffuse interstitial pulmonary fibrosis  #36 idiopathic pulmonary fibrosis cell line  #37 idiopathic pulmonary fibroses  #38 pulmonary fibroses, idiopathic  #39 idiopathic fibrosing alveolitis, chronic form  #40 fibrosing alveolitis, cryptogenic  #41 fibrocystic pulmonary dysplasia  #42 dysplasia, fibrocystic pulmonary  #43 fibrocystic pulmonary dysplasias  #44 pulmonary dysplasia, fibrocystic  #45 cryptogenic fibrosing alveolitis  #46 cryptogenic fibrosing alveolitides  #47 fibrosing alveolitides, cryptogenic  #48 pulmonary fibrosis, idiopathic  #49 usual interstitial pneumonia  #50 interstitial pneumonia, usual  #51 usual interstitial pneumonias  #52 interstitial pneumonitis, usual  #53 pneumonitides, usual interstitial  #54 pneumonitis, usual interstitial  #55 usual interstitial pneumonitides  #56 usual interstitial pneumonitis  #57 familial idiopathic pulmonary fibrosis  #58 idiopathic pulmonary fibrosis, familial  #59 OR/#27-#58  #60 #9 AND #26 AND #59 | 32 |
| **Web of Science** | #1 TS=(Non-alcoholic Fatty Liver Disease) OR AB= (NAFLD OR Nonalcoholic Fatty Liver Disease OR Fatty Liver*, Nonalcoholic OR Liver*, Nonalcoholic Fatty OR Nonalcoholic Fatty Liver* OR Nonalcoholic Steatohepatiti* OR Steatohepatiti*, Nonalcoholic)  #2 TS= (Diabetes Mellitus, Type 2) OR AB= (Diabetes Mellitus, Noninsulin-Dependent OR Diabetes Mellitus, Ketosis*Resistant OR Diabetes Mellitus, Non*Insulin Dependent OR Diabetes Mellitus, Stable OR Diabetes Mellitus, Type II OR NIDDM OR Diabetes Mellitus, Maturity*Onset OR MODY OR Diabetes Mellitus, Slow*Onset OR Type 2 Diabetes Mellitus OR Noninsulin*Dependent Diabetes Mellitus OR Diabetes Mellitus, Adult Onset)  #3 TS= (Medicine, Chinese Traditional) OR AB= (Traditional Chinese Medicine OR Chung I Hsueh OR Zhong Yi Xue OR Chinese Traditional Medicine OR Chinese Medicine, Traditional OR Traditional Tongue Diagnosis OR Tongue Diagnos*, Traditional OR Traditional Tongue Assessment*)  #4 #3 AND #2 AND #1 | 7 |
| **CNKI** | （主题=二甲双胍 OR 盐酸二甲双胍 OR 二甲基脒基胍) AND (主题=肺纤维化 OR 肺泡炎 OR 纤维化 OR Hamman-Rich综合征 OR 隐源性纤维化肺泡炎 OR 特发性肺纤维化) AND (主题=模型 OR 动物 OR 实验动物模型 OR 实验室动物模型 OR 动物实验) | 117 |
| **Wanfang**  **Data** | 主题:(二甲双胍 or 二甲基脒基胍 or 盐酸二甲双胍) and 主题:(肺纤维化 or 肺泡炎 or 纤维化 or Hamman-Rich综合征 or 特发性肺纤维化 or 隐源性纤维化肺泡炎) and 摘要:(模型 or 动物 or 实验动物模型 or 实验室动物模型 or 动物实验) | 177 |
| **VIP** | (题名或关键词=二甲双胍 OR 二甲基脒基胍 OR 盐酸二甲双胍) AND (题名或关键词=肺纤维化 OR 肺泡炎 OR 纤维化 OR Hamman-Rich综合征 OR 特发性肺纤维化 OR 隐源性纤维化肺泡炎) AND (摘要=动物 OR 模型 OR 实验动物模型 OR 实验室动物模型 OR 动物实验) | 20 |
| **CBM** | (二甲双胍 OR 二甲基脒基胍 OR 盐酸二甲双胍) AND (肺纤维化 OR 肺泡炎 OR 纤维化 OR Hamman-Rich综合征 OR 特发性肺纤维化 OR 隐源性纤维化肺泡炎) AND (模型, 动物 OR 实验动物模型 OR 实验室动物模型 OR 动物实验) | 88 |
| **MEDLINE** | TS=(Metformin) AND (TS=(Pulmonary Fibrosis) OR TS=(Hamman-Rich) OR TS=(Idiopathic Pulmonary Fibrosis)) AND (TS=(Models) OR TS=(Animal)) | 4 |

## Supplementary File S1 The PRISMA checklist of this meta-analysis

| **Section and Topic** | **Item #** | **Checklist item** | **Location where item is reported** |
| --- | --- | --- | --- |
| **TITLE** | | |  |
| Title | 1 | Identify the report as a systematic review. | 1 |
| **ABSTRACT** | | |  |
| Abstract | 2 | See the PRISMA 2020 for Abstracts checklist. | 1-2 |
| **INTRODUCTION** | | |  |
| Rationale | 3 | Describe the rationale for the review in the context of existing knowledge. | 2-3 |
| Objectives | 4 | Provide an explicit statement of the objective(s) or question(s) the review addresses. | 3 |
| **METHODS** | | |  |
| Eligibility criteria | 5 | Specify the inclusion and exclusion criteria for the review and how studies were grouped for the syntheses. | 4,34 |
| Information sources | 6 | Specify all databases, registers, websites, organisations, reference lists and other sources searched or consulted to identify studies. Specify the date when each source was last searched or consulted. | 3-4 |
| Search strategy | 7 | Present the full search strategies for all databases, registers and websites, including any filters and limits used. | 3-4 |
| Selection process | 8 | Specify the methods used to decide whether a study met the inclusion criteria of the review, including how many reviewers screened each record and each report retrieved, whether they worked independently, and if applicable, details of automation tools used in the process. | 4 |
| Data collection process | 9 | Specify the methods used to collect data from reports, including how many reviewers collected data from each report, whether they worked independently, any processes for obtaining or confirming data from study investigators, and if applicable, details of automation tools used in the process. | 4 |
| Data items | 10a | List and define all outcomes for which data were sought. Specify whether all results that were compatible with each outcome domain in each study were sought (e.g. for all measures, time points, analyses), and if not, the methods used to decide which results to collect. | 4 |
|  | 10b | List and define all other variables for which data were sought (e.g. participant and intervention characteristics, funding sources). Describe any assumptions made about any missing or unclear information. | 4 |
| Study risk of bias assessment | 11 | Specify the methods used to assess risk of bias in the included studies, including details of the tool(s) used, how many reviewers assessed each study and whether they worked independently, and if applicable, details of automation tools used in the process. | 4 |
| Effect measures | 12 | Specify for each outcome the effect measure(s) (e.g. risk ratio, mean difference) used in the synthesis or presentation of results. | 5 |
| Synthesis methods | 13a | Describe the processes used to decide which studies were eligible for each synthesis (e.g. tabulating the study intervention characteristics and comparing against the planned groups for each synthesis (item #5)). | 4 |
|  | 13b | Describe any methods required to prepare the data for presentation or synthesis, such as handling of missing summary statistics, or data conversions. | 4 |
|  | 13c | Describe any methods used to tabulate or visually display results of individual studies and syntheses. | 4-5 |
|  | 13d | Describe any methods used to synthesize results and provide a rationale for the choice(s). If meta-analysis was performed, describe the model(s), method(s) to identify the presence and extent of statistical heterogeneity, and software package(s) used. | 5 |
|  | 13e | Describe any methods used to explore possible causes of heterogeneity among study results (e.g. subgroup analysis, meta-regression). | 5 |
|  | 13f | Describe any sensitivity analyses conducted to assess robustness of the synthesized results. | 5 |
| Reporting bias assessment | 14 | Describe any methods used to assess risk of bias due to missing results in a synthesis (arising from reporting biases). | 5 |
| Certainty assessment | 15 | Describe any methods used to assess certainty (or confidence) in the body of evidence for an outcome. | none |
| **RESULTS** | | |  |
| Study selection | 16a | Describe the results of the search and selection process, from the number of records identified in the search to the number of studies included in the review, ideally using a flow diagram. | 5-6 |
|  | 16b | Cite studies that might appear to meet the inclusion criteria, but which were excluded, and explain why they were excluded. | 5 |
| Study characteristics | 17 | Cite each included study and present its characteristics. | 5-6 |
| Risk of bias in studies | 18 | Present assessments of risk of bias for each included study. | 7 |
| Results of individual studies | 19 | For all outcomes, present, for each study: (a) summary statistics for each group (where appropriate) and (b) an effect estimate and its precision (e.g. confidence/credible interval), ideally using structured tables or plots. | 7-9 |
| Results of syntheses | 20a | For each synthesis, briefly summarise the characteristics and risk of bias among contributing studies. | 7-9 |
|  | 20b | Present results of all statistical syntheses conducted. If meta-analysis was done, present for each the summary estimate and its precision (e.g. confidence/credible interval) and measures of statistical heterogeneity. If comparing groups, describe the direction of the effect. | 7-9 |
|  | 20c | Present results of all investigations of possible causes of heterogeneity among study results. | 7-9 |
|  | 20d | Present results of all sensitivity analyses conducted to assess the robustness of the synthesized results. | 7-9 |
| Reporting biases | 21 | Present assessments of risk of bias due to missing results (arising from reporting biases) for each synthesis assessed. | 7-9 |
| Certainty of evidence | 22 | Present assessments of certainty (or confidence) in the body of evidence for each outcome assessed. | none |
| **DISCUSSION** | | |  |
| Discussion | 23a | Provide a general interpretation of the results in the context of other evidence. | 9-14 |
|  | 23b | Discuss any limitations of the evidence included in the review. | 13 |
|  | 23c | Discuss any limitations of the review processes used. | 13 |
|  | 23d | Discuss implications of the results for practice, policy, and future research. | 14 |
| **OTHER INFORMATION** | | |  |
| Registration and protocol | 24a | Provide registration information for the review, including register name and registration number, or state that the review was not registered. | 3 |
|  | 24b | Indicate where the review protocol can be accessed, or state that a protocol was not prepared. | 3 |
|  | 24c | Describe and explain any amendments to information provided at registration or in the protocol. | none |
| Support | 25 | Describe sources of financial or non-financial support for the review, and the role of the funders or sponsors in the review. | 14 |
| Competing interests | 26 | Declare any competing interests of review authors. | 14 |
| Availability of data, code and other materials | 27 | Report which of the following are publicly available and where they can be found: template data collection forms; data extracted from included studies; data used for all analyses; analytic code; any other materials used in the review. | 21 |

*From:*  Page MJ, McKenzie JE, Bossuyt PM, Boutron I, Hoffmann TC, Mulrow CD, et al. The PRISMA 2020 statement: an updated guideline for reporting systematic reviews. BMJ 2021;372:n71. doi: 10.1136/bmj.n71

For more information, visit: <http://www.prisma-statement.org/>

15：Not applicable.

22：Not applicable.

24C：There is no amendment to information provided at registration or in the protocol in this review.

## Supplementary Table S2 Basic characteristics of the included studies

| **Study** | **Animals Type** | **Sample size A/C** | **Interventions**  **A/C** | **Death** | **Anesthetics** | **Ethics Statement** | **Location** | **Result Index** |
| --- | --- | --- | --- | --- | --- | --- | --- | --- |
| Farhood, et al. (2019) | male mice | 10/10 | Post-radiation MET intervention/radiation | / | Ketamine and Xylazine at 20 and 5 mg/kg | available | Iran | 1, 5 |
| Cheng, et al. (2021) | male mice | 6/6/6/6 | 100mg/kg MET + Silica/300mg/kg MET + Silica(2W)/300mg/kg MET + Silica (4W)/silica | / | / | available | Nanjing,China | 1, 3, 4, 6 |
| Gamad, et al. (2018) | adult male Wistar rats | 8/8/8/8 | 125mg/kg/d MET + Bleomycin /250mg/kg/d MET + Bleomycin /500mg/kg/d MET + Bleomycin / Bleomycin | / | Pentobarbitone sodium (60 mg/kg) | available | New Delhi, India | 1, 2, 3, 6, 7, 8, 9 |
| Gu, et al.（2021） | male wild type mice | 20/20 | 65mg/kg MET + Bleomycin / Bleomycin | / | / | available | Hanzhong, China | 1, 6, 7 |
| Hao, et al. （2019） | SD male Rats | 12/12/12 | Bleomycin + MET /Bleomycin | 3/2/3 | Pentobarbital sodium | / | Anhui, China | 2, 4, 6, 8, 9, 10 |
| Huang（2015） | SD male Rats | 10/10 | Bleomycin + MET /Bleomycin | / | Pentobarbital sodium | / | Chongqing, China | 3 |
| Jian (2017) | SD male Rats | 6/6 | Bleomycin + MET /Bleomycin | / | Chloral hydrate 10% intraperitoneal injection | / | Zunyi, China | 1, 2, 3, 5, 7 |
| Jiang (2016) | SD male Rats | 4/4 | Bleomycin + MET /Bleomycin | / | Chloral hydrate 10% intraperitoneal injection | / | Zunyi, China | 1, 2, 3, 5 |
| Li, et al. (2021) (A) | Wistar male rats | 8/8 | Silica particles +MET / Silica particles | / | Pentobarbital sodium | available | Shandong, China | 1, 2, 4, 5 |
| Li, et al. (2021) (B) | Wistar male rats | 8/8 | Silica particles +MET / Silica particles | / | / | available | Shandong, China | 1, 2, 3, 4, 5 |
| Rangarajan, et al. (2018) | C57BL/6 male mice | 3/3 | Bleomycin +MET /Bleomycin +saline | / | Isoflurane | available | Birmingham, USA | 2, 3, 4, 7 |
| Yahyapour, et al. (2019) | male NMRI mice | 10/10 | 100mg/kg MET+ radiation/radiation | / | Ketamine (20mg/kg) and Xylazine (5mg/kg) | / | Jiroft, Iran | 1 |
| Choi, et al. (2016) | female C57BL/6 mice | 14/14/14 | 50mg/kg MET+ Bleomycin /100mg/kg MET + Bleomycin / Bleomycin | 3/1/5 | / | / | Seoul, Korea | 1, 2, 6, 10 |
| Wang Y, et al. (2017) | adult female SD RatS | 5/5 | 200 mg/kg/d MET + radiation/radiation | / | Pentobarbital sodium | available | Wuhan, China | 1, 2, 3, 6, 7, 8 |
| Wang, et al. (2020) | male SD rats | 6/6 | 300mg/kg MET + Bleomycin / Bleomycin | / | Chloral hydrate | available | Chongqing, China | 4, 6, 8, 9 |
| Wang J, et al. (2017) | female SD rats | 5/5 | 200mg/kg MET + radiation/ radiation | / | / | available | Wuhan, China | 1, 2, 3, 6, 7, 8 |
| Wu (2018) | male SD rats | 8/8 | 500mg/kg MET + Bleomycin /Bleomycin | 1/2 | Chloral hydrate | available | Zunyi, China | 1, 2, 3, 5, 7, 10 |
| Xiao, et al. (2018) | mice | 10/10 | 200mg/kg MET + Bleomycin /Bleomycin | 3/6 | Pentobarbital sodium | / | Beijing, China, | 1, 2, 3, 10 |
| [Xiao et al. (2020](https://www.ncbi.nlm.nih.gov/pmc/articles/PMC7137034/)) | male C57BL/6J mice | 10/10 | 200 mg/kg/d MET + 300 mg/kg/d Pirfenidone + Bleomycin /300 mg/kg/d Pirfenidone +Bleomycin | / | / | / | Beijing, China, | 3, 4, 6 |

***NOTE:*** A/C, Experimental group/control group; 1, Lung fibrosis score; 2, TGF-β; 3, HYP; 4, α-SMA; 5, Inflammation score; 6, Collagen I; 7, AMPK; 8, Smad; 9, ERK; 10, Mortality (Experimental group/control group).

**Table 3.** Modeling and drug administration in the included studies

## Supplementary Table S3 Modeling and Drug Administration in the Included Studies

| Study | Moulding method | Therapeutic drug | Drug concentration | Dosing time | Drug Manufacturers | Dosage | Frequency | Route of administration |
| --- | --- | --- | --- | --- | --- | --- | --- | --- |
| Farhood, et al. (2019) | Radiation | MET (98%) | 3mg /ml | 2W | Tehran Chemie | 100mg/kg | 5 per week | oral administration |
| Cheng, et al. (2021) | Silica | MET | / | 2W, 4W | Beyotime | 100mg/kg 300mg/kg | every day | intragastrical administration |
| Gamad, et al. (2018) | BLM | MET | / | 5W | Cipla | 125mg/kg 250mg/kg 500mg/kg | every day | intraperitoneal injection |
| Gu, et al.（2021） | BLM | MET | / | 2W,4W | Sigma-Aldrich | 65mg/kg | every second day | intraperitoneal injection |
| Hao, et al. （2019） | BLM | MET hydrochloride tablets | / | 4W | Shanghai Sine | 100mg/kg 300mg/kg | every day | tracheal instillation |
| Huang（2015） | BLM | MET | / | 3W | Sigma-Aldrich | 300mg/kg | every day | intragastrical administration |
| Jian (2017) | BLM | MET | 0.5g in 10ml | 2W | Sino-American Shanghai Squibb | 250mg/kg | every day | intragastrical administration |
| Jiang (2016) | BLM | MET | 0.5g in 10ml | 2W | Sino-American Shanghai Squibb | 250 mg/Kg | every day | intragastrical administration |
| Li, et al. (2021) (A) | Silica | MET | / | 4W | Sino-American Shanghai Squibb | 100 mg / kg  200 mg / kg  400mg / kg | every day | intragastrical administration |
| Li, et al. (2021) (B) | Silica | MET hydrochloride tablets | / | 4W | Sino-American Shanghai Squibb | 100 mg / kg  200 mg / kg  400mg / kg | every day | intraperitoneal injection |
| Rangarajan, et al. (2018) | BLM | MET hydrochloride tablets | / | 18 d | Sigma-Aldrich | 65mg/kg | every other day | intraperitoneal injection |
| Yahyapour, et al. (2019) | Radiation | MET | 3 mg/ml | 80d | Tehran Chemie | 100 mg / kg | every day | intragastric administration |
| Choi, et al. (2016) | BLM | MET | / | 10d,21d | Sigma-Aldrich | 50mg/kg 100mg/kg | every day | oral administration |
| Wang Y, et al. (2017) | Radiation | MET | / | 2W | Sigma-Aldrich | 200 mg/kg | every day | intraperitoneal injection |
| Wang, et al. (2020) | BLM | MET | / | 30d | / | 300mg/kg | every day | intraperitoneal injection |
| Wang J, et al. (2017) | Radiation | MET | / | 2W | / | 200 mg/kg | every day | intraperitoneal injection |
| Wu (2018) | BLM | MET | 0.5g in 10ml | 3W | American Shanghai Squibb | 500mg/kg | every day | oral administration |
| Xiao, et al. (2018) | BLM | MET | 12.5mg/ml | 2W | American Shanghai Squibb | 200 mg/kg | every day | oral administration |
| [Xiao et al. (2020](https://www.ncbi.nlm.nih.gov/pmc/articles/PMC7137034/)) | BLM | MET | / | 2W | / | 200 mg/kg | every day | oral administration |

***NOTE:*** BLM, bleomycin tracheal instillation; Silica, silica particles.
